# ST6Gal-I–mediated sialylation of the epidermal growth factor receptor modulates cell mechanics and enhances invasion

**DOI:** 10.1016/j.jbc.2022.101726

**Published:** 2022-02-12

**Authors:** Tejeshwar C. Rao, Reena R. Beggs, Katherine E. Ankenbauer, Jihye Hwang, Victor Pui-Yan Ma, Khalid Salaita, Susan L. Bellis, Alexa L. Mattheyses

**Affiliations:** 1 Department of Cell, Developmental, and Integrative Biology, University of Alabama at Birmingham, Birmingham, Alabama, USA; 2 Department of Chemistry, Emory University, Atlanta, Georgia, USA

**Keywords:** glycosylation, ST6Gal-I, sialyltransferase, EGFR signaling, force probes, cancer, cell mechanics, AKT, Akt serine/threonine kinase, BrdU, 5-bromo-2′-deoxyuridine, BSA, bovine serum albumin, EGFR, epidermal growth factor receptor, ERK, extracellular-signal-regulated kinase, EV, empty vector, FA, focal adhesions, FasR, fas cell surface death receptor, FBS, fetal bovine serum, Kd, knockdown, OE, overexpression, PI3K, phosphoinositide 3-kinase, PS, penicillin-streptomycin, RICM, reflective interference contrast microscopy, SNA, Sambucus nigra, ShC, shRNA control, STAT, signal transducer and activator of transcription, TGT, tension gauge tether, TIRF, total internal reflection fluorescence, TNFR, tumor necrosis factor receptor, T_tol_, tension tolerance

## Abstract

Heterogeneity within the glycocalyx influences cell adhesion mechanics and signaling. However, the role of specific glycosylation subtypes in influencing cell mechanics *via* alterations of receptor function remains unexplored. It has been shown that the addition of sialic acid to terminal glycans impacts growth, development, and cancer progression. In addition, the sialyltransferase ST6Gal-I promotes epidermal growth factor receptor (EGFR) activity, and we have shown EGFR is an ‘allosteric mechano-organizer’ of integrin tension. Here, we investigated the impact of ST6Gal-I on cell mechanics. Using DNA-based tension gauge tether probes of variable thresholds, we found that high ST6Gal-I activity promotes increased integrin forces and spreading in Cos-7 and OVCAR3, OVCAR5, and OV4 cancer cells. Further, employing inhibitors and function-blocking antibodies against β1, β3, and β5 integrins and ST6Gal-I targets EGFR, tumor necrosis factor receptor, and Fas cell surface death receptor, we validated that the observed phenotypes are EGFR-specific. We found that while tension, contractility, and adhesion are extracellular-signal-regulated kinase pathway-dependent, spreading, proliferation, and invasion are phosphoinositide 3-kinase-Akt serine/threonine kinase dependent. Using total internal reflection fluorescence microscopy and flow cytometry, we also show that high ST6Gal-I activity leads to sustained EGFR membrane retention, making it a key regulator of cell mechanics. Our findings suggest a novel sialylation-dependent mechanism orchestrating cellular mechanics and enhancing cell motility *via* EGFR signaling.

Mechanical forces are key regulators of cell structure and function, playing a crucial role in many processes including mitosis, apoptosis, adhesion, and migration (1, 2). Studying the mechanical properties of cells is vital to decode the underlying mechanisms by which cells sense environmental stimuli and translate them into biochemical cues that influence cellular outcomes (3, 4). At the cell-extracellular matrix interface lies the glycocalyx, a complex mixture of glycoproteins, glycolipids, and free glycans that act as a cushion around living cells (5, 6). Dysregulation of cell or tissue function, similar to that observed in cancers, results in a substantial increase in the glycocalyx, which directly alters integrin-mediated signaling, membrane receptor functions, and cell–matrix mechanical interactions (7, 8, 9). Cell-surface receptors can be modified with specific sugars, which can change how they communicate external cues and translate them into intracellular signals (10). While the glycocalyx plays a key role in influencing cell and tissue mechanics (11, 12, 13), the molecular details of how particular sugar modifications on specific proteins mediate the mechanics of cell–matrix interactions remains largely overlooked.

Amongt the myriad membrane receptor signaling pathways involved in maintaining cell-matrix homeostasis, the adhesion receptor integrin and epidermal growth factor receptor (EGFR) have robust synergy (14, 15). While the importance and underlying biochemical mechanisms of this crosstalk are established, the observed effects are attributed to molecules downstream from the receptors, away from the plasma membrane (16, 17, 18, 19, 20, 21). Recently, our laboratory identified a novel role for activated EGFR as a ‘mechano-organizer’ where it modulates the mechanical threshold for integrin activation (22). We proposed that EGFR and integrin act as a joint-sensing apparatus, similar to a signaling rheostat, to tune the cell’s mechanical response and facilitate cell spreading *via* organization of integrin tension and maturation of focal adhesions (FAs) (22).

Over half of mammalian proteins are glycosylated, which can impact their structure and function. Without the correct sugar modifications, many proteins misfold or become unstable. Various subtypes of glycosylation provide diverse cues to supplement the broad range of biological functions that proteins perform. One specific form of glycosylation, sialylation, employs enzymes called sialyltransferases which transfer sialic acid residues from cytidine monophosphate N-acetylneuraminic acid to N- or O-linked glycan chains. The sialic acid is covalently attached to the underlying glycan chain *via* distinct glycosidic linkages (α2,3, α2,6, or α2,8). As a result, sialyltransferases are classified into four main groups depending on the type of glycosidic bond they generate: ST3Gal1-6 (α2,3 sialyltransferases), ST6Gal1-2 and ST6GalNAc1-6 (α2,6 sialyltransferases), and ST8Sia1-6 (α2,8 sialyltransferases) (23). These distinct glycosidic linkages result in fundamental structural differences, which can directly alter protein function. While several sialyltransferase enzymes have been implicated in cancer, ST6Gal-I has garnered increased attention in recent literature. ST6Gal-I is upregulated across different cancer types, including breast, gliomas, pancreatic, prostate, and ovarian cancer, and plays a fundamental role in tumor progression, epithelial-to-mesenchymal transition, and metastasis (24, 25, 26, 27, 28).

Our group previously demonstrated that ligand-dependent EGFR activity was robustly increased by α2,6 sialylation of N-glycans (29, 30). ST6Gal-I mediated sialylation of β1 integrin has also been shown to drive tumor cell migration and invasion (31). Increased sialylation alters the oligomerization of membrane receptors including CD45, PECAM, and EGFR (32, 33, 34). ST6Gal-I is the sialyltransferase that catalyzes the addition of α2,6-linked sialic acids onto subterminal galactose residues of lactosaminic chains of N-glycans (Galβ1,4GlcNAc) (35, 36, 37). Sialylation by ST6Gal-I is a specific glycomodification, which has been demonstrated to influence membrane-receptor function. Given the role of activated EGFR in regulating integrin mechanics, we wanted to explore how sialylation of EGFR influences integrin-dependent adhesion and cell mechanics.

Clinically, increased glycoprotein sialylation has been associated with carcinogenesis, and ST6Gal-I promotes vital cancer hallmarks such as self-renewal, invasiveness, proliferative potential, and resistance to cell death (28). Mechanical changes in cells and tissues also contribute to malignancy and metastasis, but the underlying mechanisms by which these changes promote cancer remain understudied (21, 38, 39, 40, 41, 42, 43). To delineate the functional role of ST6Gal-I-mediated sialylation on EGFR-integrin crosstalk–dependent mechanical phenotypes, we employed DNA-based tension gauge tether (TGT) surfaces. Using high resolution total internal reflection fluorescence (TIRF) microscopy, we show that ST6Gal-I mediated sialylation of EGFR in Cos-7 cells enhances EGF-driven mechanical changes including increased integrin tension, FA maturation, cell spreading, and migration. We validate that these phenotypes are driven by EGFR signaling with pharmacological, biochemical, and classical cancer biology assays. We use inhibitors to identify which signaling cascades downstream of EGFR regulate the mechanical outcomes. We find changes in cell mechanics, and FA maturation are driven by the extracellular-signal-regulated kinase (ERK) signaling pathway, while the enhancement of cell spreading, migration, and invasion are driven by phosphoinositide 3-kinase-Akt serine/threonine kinase (PI3K–AKT) signaling pathway. These results assign specific mechanistic roles to downstream signaling cascades in coordinating distinct cellular responses following EGFR activation. Finally, we show that EGFR is retained at the plasma membrane in cells expressing ST6Gal-I compared to controls. These results highlight a crucial mechanism where ST6Gal-I is a novel regulator of mechanosignaling through EGFR membrane retention and activity.

## Results

### ST6Gal-I regulates EGF-induced EGFR activation in Cos-7 cells

**Figure 1:**
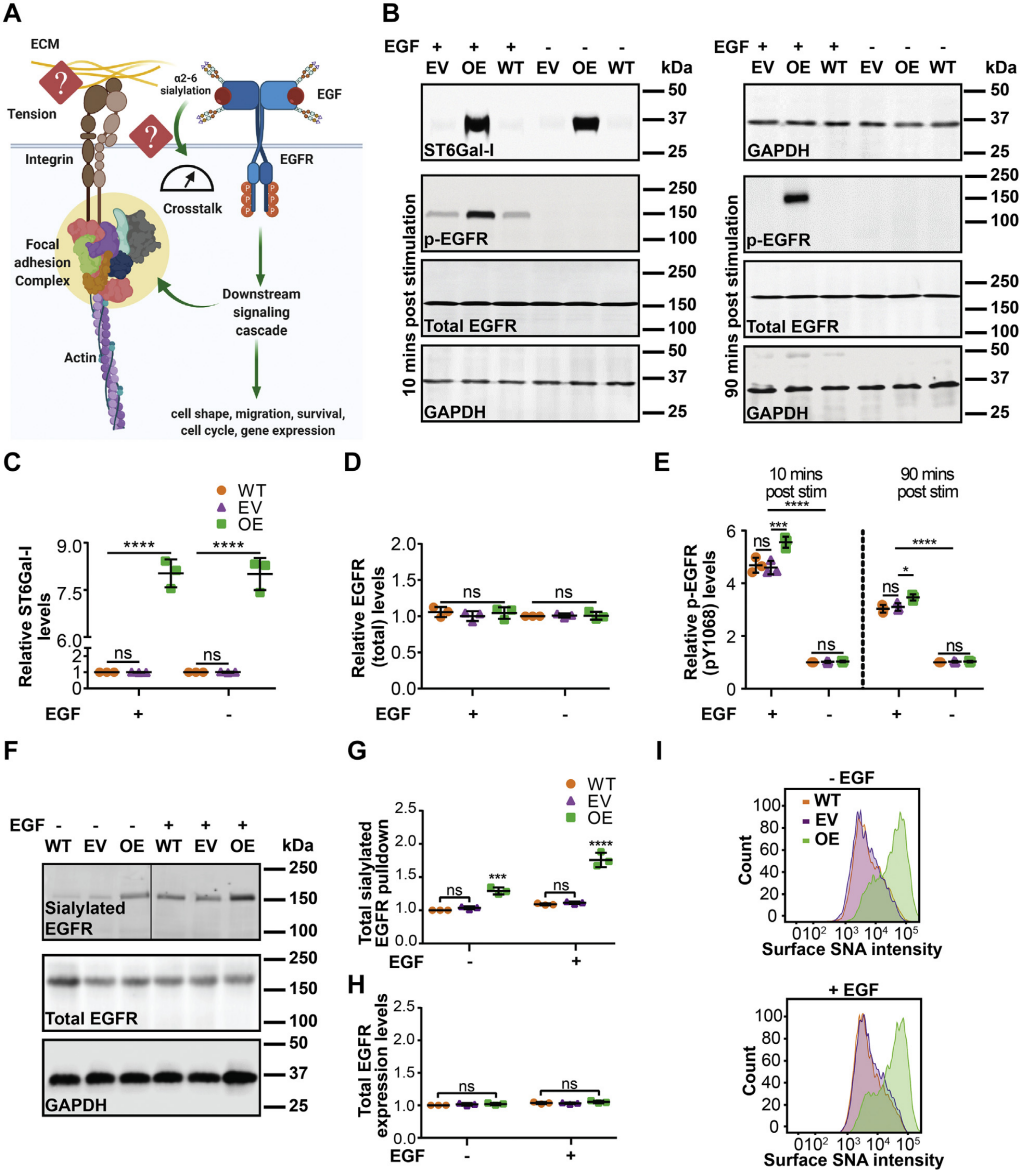
**Epidermal growth factor receptor sialylation is enhanced in Cos-7 cells with ST6Gal-I overexpression.***A*, schematic of EGFR-integrin crosstalk and the gap in knowledge as to how EGFR glycosylation may regulate cell mechanics. *B*, representative immunoblots of ST6Gal-I, total EGFR, and pEGFR from Cos-7 cells stably transduced with lentivirus encoding human ST6Gal-I (OE) or empty vector (EV) and WT Cos-7 controls with (10 min, 90 min) or without EGF stimulation. GAPDH was used as the loading control. *C*–*E*, quantification of (*C*) ST6Gal-I, (*D*) total EGFR, and (*E*) p-EGFR normalized to WT cells without EGF treatment. (mean ± SD, n = 3 independent experiments; ^ns^p > 0.05, ∗*p* < 0.05, ∗∗∗*p* < 0.001, ∗∗∗∗*p* < 0.0001 by one-way ANOVA with Tukey’s test). *F*, immunoblots of α2,6 sialylated EGFR and total EGFR in WT, EV, and OE Cos-7 cells with or without EGF stimulation. GAPDH was used as the loading control. *G* and *H*, densitometric analysis normalized with respect to WT without EGF stimulation quantifying the levels of (*G*) α2,6 sialylated EGFR and (*H*) total EGFR. (mean ± SD; n = 3 independent experiments, ^ns^p > 0.05, ∗*p* < 0.05 by one-way ANOVA with Tukey’s test). *I*, SNA staining of α2,6 surface sialylation in WT, EV, and ST6Gal-I OE cells measured by flow cytometry without or with EGF (10 min) stimulation.

Epidermal growth factor receptor can coordinate with integrins to regulate cell adhesion and growth (44, 45). A cell’s ability to respond to extracellular signals is intimately connected to its ability to pull against ligands in the extracellular matrix, which is conferred by the tensional and architectural organization of the cytoskeleton (46, 47, 48). Results from our lab demonstrate that ligand-dependent EGFR signaling attenuates the threshold for outside-in mechanical activation of integrins and enhances FA maturation (22). Our research has also shown that ST6Gal-I mediated α2,6 sialylation promotes EGFR activity (29, 30). Therefore, we wanted to test if increased ST6Gal-I activity could alter cell mechanics and morphological outcomes during adhesion and growth (Fig. 1*A*). To evaluate the role of ST6Gal-I mediated sialylation in cell mechanics, the Cos-7 cell line, which has negligible ST6Gal-I expression, was transduced with a lentivirus to stably overexpress ST6Gal-I (OE). This led to increased ST6Gal-I expression relative to empty vector (EV) or WT controls as validated by Western blot (Fig. 1, *B* and *C*). We found no significant difference in total EGFR protein levels in OE and EV cells (Fig. 1, *B* and *D*). To see if ST6Gal-I OE impacted EGFR activation, we used an antibody against phosphorylated (activated) EGFR (p-Tyr 1068) (Fig. 1*B*). At 10 and 90 min following EGF stimulation, ST6Gal-I OE cells had increased EGFR activation relative to EV or WT cells, in agreement with previous work (Fig. 1*E*) (29, 30). ST6Gal-I OE resulted in an increase in α2,6 sialylation of EGFR compared with EV or WT controls, as measured by immunoblotting following pull-down by the Sambucus nigra (SNA) lectin which specifically recognizes α2,6 sialic acids (49) (Fig. 1, *F* and *G*). The total EGFR protein levels were comparable in WT, EV, and OE cells (Fig. 1, *F* and *H*). To verify that overexpression of ST6Gal-I led to a concomitant increase in cell surface sialylation, WT, EV, and ST6Gal-I OE Cos-7 cells were labeled with SNA, with or without EGF stimulation, and quantified by flow cytometry (Fig. 1*I*). ST6Gal-I OE Cos-7 cells had a marked increase in α2,6 sialylation of cell surface proteins, regardless of EGF stimulation.

### ST6Gal-I–mediated EGFR sialylation modulates cell spreading, integrin tension, and FA organization with EGF stimulation

**Figure 2:**
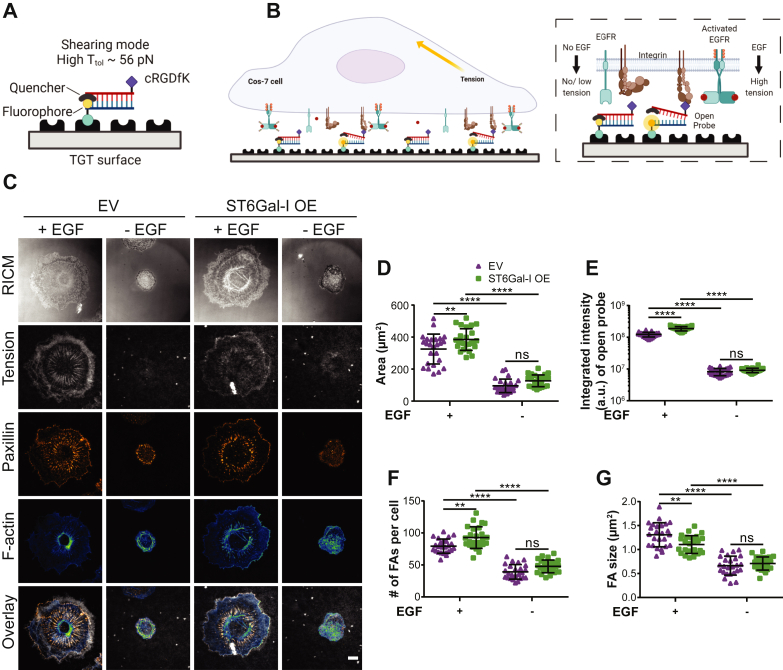
**ST6Gal-I regulates cell spreading, integrin tension, and FA maturation in an EGF-dependent manner.***A*, schematic of the 56 pN TGT probe. *B*, illustration of the cell-TGT surface contact zone highlighting the interaction of integrins with cRGDfK in the presence or absence of EGF. *C*, images of empty vector (EV) and ST6Gal-I overexpressing (OE) Cos-7 cells on a 56 pN TGT surface 90 min postplating in the presence or absence of EGF (RICM, integrin tension - *grayscale*, paxillin - *orange hot*, and actin - *green blue*; scale bar 10 μm). *D*–*G*, quantification of the (*D*) spread area (EV, OE cells with EGF: 325.3 ± 93.2 μm^2^, 384.7 ± 67.7 μm^2^; without EGF: 95.7 ± 42.3 μm^2^, 126.9 ± 36.8 μm^2^), (*E*) integrated intensity of open probes (EV, OE cells with EGF: 1.11∗10^8^ ± 3.2∗10^7^ a.u., ±1.46∗10^8^ ± 2.4∗10^7^ a.u.; without EGF: 8.9∗10^6^ ± 4.8∗10^6^ a.u., 9.92∗10^6^ ± 3.8∗10^6^ a.u.), (*F*) number of focal adhesions (FAs) per cell (EV, OE cells with EGF: 79.4 ± 11.0, 92.8 ± 16.9; without EGF: 39.2 ± 11.4, 47.8 ± 9.97), and (*G*) FA size (EV, OE cells with EGF: 1.31 ± 0.3 μm^2^, 1.10 ± 0.2 μm^2^; without EGF: 0.66 ± 0.2 μm^2^, 0.71 ± 0.1 μm^2^). (Mean ± SD, n = 25 cells across three independent experiments; ^ns^p > 0.05, ∗∗*p* < 0.01, ∗∗∗∗*p* < 0.0001 by one-way ANOVA with Tukey’s test).

We next wanted to investigate if ST6Gal-I influenced the morphometric and mechanical responses to EGF stimulation we previously reported. For this we employed ‘turn-on’ TGT probes presenting the integrin ligand cyclic Arg-Gly-Asp-Phe-Lys (cRGDfK) (22, 50, 51, 52). cRGDfK is highly selective for αVβ3 integrin with a smaller affinity for α5β1 integrin (53, 54, 55, 56). The TGT probe design consists of a DNA duplex immobilized on the coverslip surface using the bottom strand, while the top strand displays the cRGDfK ligand. The bottom strand is labeled with a fluorophore and the top strand with a quencher, so while the duplex is intact, there is minimal fluorescence (Figs. 2, *A* and *B* and S1). Upon binding to the ligand, if the integrin applies a tension larger than the tension tolerance (T_tol_) of the probe, the DNA duplex will dissociate and generate a fluorescent signal (Fig. 2*B*). Any TGT probes that are not ruptured by a mechanical force will remain nonfluorescent due to quenching. Tension gauge tethers allow us to quantitatively map the spatial distribution of integrin-generated forces that exceed the T_tol_ threshold. Additionally, they modulate the tension that can be supported by the underlying substrate. To cover the wide range of tensions experienced by integrins, we employed TGTs with similar chemical compositions but different geometries- ‘unzipping’ (T_tol_ = 12 pN, lower tension threshold) and ‘shearing’ (T_tol_ = 56 pN, higher tension threshold) (22, 51, 57, 58). Note that these two probes have identical sequences and thermal melting temperatures and differ only in their mechanical stability.

We first investigated the effect of EGF stimulation on cell adhesion and spreading in ST6Gal-I OE cells compared to EV controls. The cells were plated on TGT surfaces for 90 min, fixed, labeled with markers for FAs and actin, and imaged using reflective interference contrast microscopy (RICM) and TIRF microscopy (Fig. 2*C*). Reflective interference contrast microscopy reveals the cell-substrate contact region, or the cell footprint, and reports on cell spreading. Total internal reflection fluorescence microscopy specifically illuminates a ∼100 nm region in the sample adjacent to the coverslip, allowing imaging of open TGT probes and plasma membrane proximal FA proteins and actin, while eliminating out of focus fluorescence from within the cell. In agreement with our previous work, EGF stimulation lowered the tension threshold required for a cell to spread. Additionally, cell spreading was significantly increased in ST6Gal-I OE cells on the 56 pN TGT surfaces compared to EV controls when stimulated with EGF (Fig. 2, *C* and *D*). ST6Gal-I OE also enhanced the cell’s ability to spread on substrates of lower tension threshold (12 pN) leading to significantly larger spread areas (Fig. S2, *A* and *B*). We further evaluated cell mechanical changes by quantifying the integrated intensity of open TGT probes and found ST6Gal-I OE led to increased integrin tension on both the 56 and 12 pN TGT surfaces when stimulated with EGF (Figs. 2*E* and S2*C*).

Next, we evaluated the impact of ST6Gal-I OE on FAs. ST6Gal-I OE increased the number of FAs per cell on both TGT surfaces with EGF stimulation (Figs. 2*F* and S2*D*). Focal adhesion size is an indicator of maturity, where FAs larger than 1 μm^2^ represent more mature adhesions and those between 0.2 and 0.6 μm^2^ represent nascent adhesions (59). Interestingly, the effect of ST6Gal-I OE on FA size varied based on the TGT tension threshold. While FAs in OE cells were larger than in EV cells on the 12 pN TGT surface, they were significantly smaller on the 56 pN surface (Figs. 2*G* and S2*E*). Despite the reduction in FA size, the footprint of ST6Gal-I OE cells was increased indicating FA maturity does not directly correlate with the cell area. The observed differences between the 12 and the 56 pN TGT surfaces could be in part due to the modulation of the underlying TGT tension threshold experienced by the cell.

**Figure 3:**
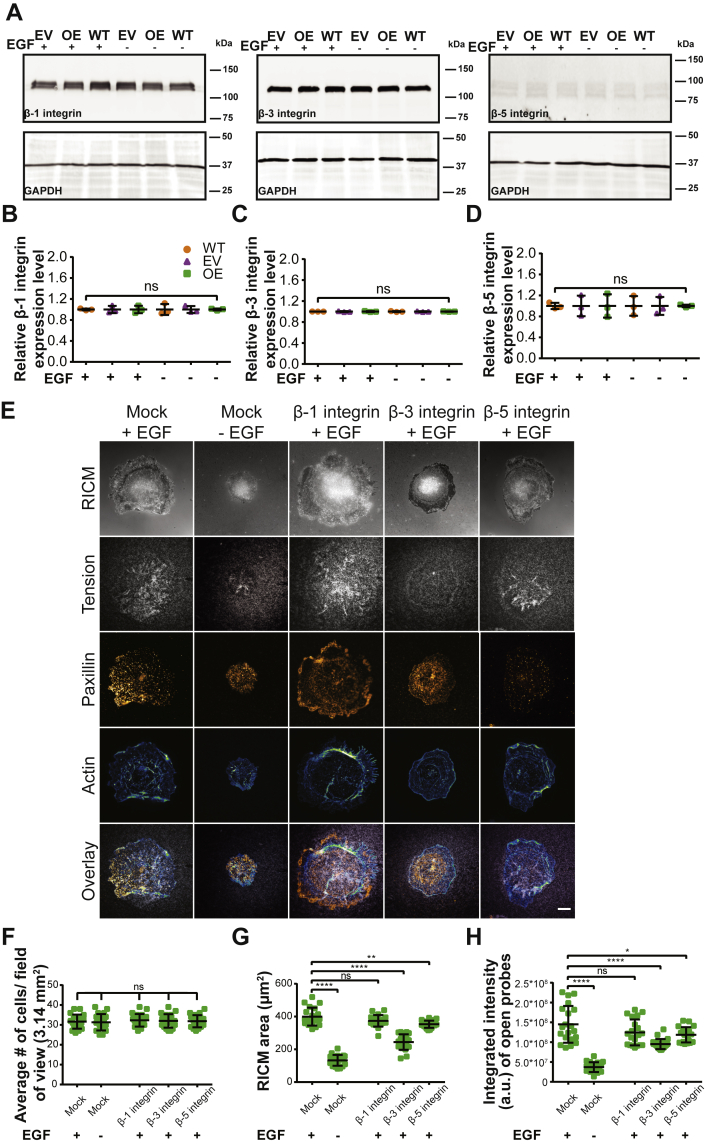
**Mechanical outcomes are modulated by specific β-integrin subtypes.***A*, representative immunoblots of β1, β3, and β5 integrin from WT, empty vector (EV), and ST6Gal-I overexpressing (OE) Cos-7 cells with (10 min) or without EGF stimulation. *B*–*D*, quantification of (*B*) β1 integrin, (*C*) β3 integrin, and (*D*) β5 integrin expression normalized to WT cells without EGF. (mean ± SD, n = 3 independent sets of experiments; ^ns^p > 0.05 by one-way ANOVA with Tukey’s test). *E*, images of mock [+ (with) and – (without) EFG] and β1, β3, and β-5 integrin-blocking antibody + EGF treated ST6Gal-I OE Cos-7 cells on a 56 pN TGT surface 90 min postplating (RICM, integrin tension - *grayscale*, paxillin - *orange hot*, and actin - *green blue*; scale bar 10 μm). *F*–*H*, quantification of the (*F*) average number of cells per field of view (with EGF: mock, 32 ± 3.5; β1, 32 ± 3.1; β3, 32 ± 3.4; β5, 32 ± 3.2; without EGF: mock, 31 ± 4.1), (*G*) cell spread area (with EGF: mock, 399.5 ± 54.8 μm^2^; β1, 374.0 ± 35.3 μm^2^; β3, 244.4 ± 47.2 μm^2^; β-5, 353.0 ± 21.7 μm^2^; without EGF: mock, 132.8 ± 33.2 μm^2^), and (*H*) integrated intensity of open probes (with EGF: mock, 1.5∗10^8^ ± 4.6∗10^7^ a.u.; β1, 1.2∗10^8^ ± 3.3∗10^7^ a.u.; β3, 9.5∗10^7^ ± 1.3∗10^7^ a.u.; β5, 1.2∗10^8^ ± 1.9∗10^7^ a.u.; without EGF: mock, 3.7∗10^7^ ± 1.2∗10^7^ a.u.) (mean ± SD, n = 25 cells across three independent experiments; ^ns^p > 0.05, ∗*p* < 0.05, ∗∗*p* < 0.01, ∗∗∗∗*p* < 0.0001 by one-way ANOVA with Tukey’s test).

One possible route for ST6Gal-I mediated mechanical changes is through integrins. Our previous research suggested that ST6Gal-I-mediated α2,6 sialylation represents an important mechanism for β1, but not β3 or β5 integrins (31). Additionally, the ligand on our TGT probes (cRGDfK) is highly selective for αVβ3 integrins with a low affinity for α5β1 integrin. Therefore, we decided to evaluate the expression profiles for β1, β3, and β5 integrins in Cos-7 cells. We found no significant difference in total β1, β3, or β5 integrin protein expression levels in ST6Gal-I OE and EV cells compared to WT Cos-7 controls (Fig. 3, *A*–*D*). β1 and β3 were the two major isoforms expressed in Cos-7 cells, and there was very little β5 expression (Fig. 3*A*). To see if either β1 or β3 integrin were a substrate for ST6Gal-I mediated α2,6 sialylation, we conducted an SNA pulldown followed by immunoblotting of WT, EV, and ST6Gal-I OE cells. This showed that β1 but not β3 integrin was α2,6 sialylated in ST6Gal-I OE Cos-7, in agreement with our previous findings (Fig. S3) (31). To further examine the roles of these integrins in regulating EGF-stimulated cell mechanics, we used function blocking antibodies against β1, β3, or β5 integrin. ST6Gal-I OE cells were preincubated with antibody for 20 min prior to plating on 56 pN TGT surfaces. Blocking individual β-subunits did not alter the total number of cells that attached to the surface compared to the mock (DMSO)-treated controls (Fig. 3*F*). In terms of the mechanical outcomes, β3-blocking antibody had the biggest impact on both cell spreading and integrin tension generation, followed by β5 integrin (Fig. 3, *E*, *G*, and *H*). β1-blocking antibody had no significant effect on either the spread area or the integrated integrin tension. These results reflect the high affinity of the TGT ligand cRGDfK for αVβ3 integrin. It should be noted that the changes in mechanical outcomes were EGF-stimulation dependent. Further evidence that sialylation of integrins does not contribute to the outcomes presented here is that no significant change in cell spread area or integrated integrin tension was observed in ST6Gal-I OE cells compared to EV controls without EGF stimulation (Fig. 2, *D* and *E*). Overall, we conclude that while β1 integrins are sialylated by ST6Gal-I, they do not participate in changing cell mechanics on cRGDfK TGT surfaces and the EGF-stimulated increase in cell mechanics presented here cannot be attributed to integrin sialylation.

To investigate if the enhanced mechanics could be driven by sialylation in general, we evaluated the role of ST3Gal-4 on cell mechanics. Unlike ST6Gal-I that catalyzes α2,6 sialic acid addition, ST3Gal-4 is a principal sialyltransferase responsible for α2,3 sialic acid addition to the termini N- or O-glycans (60). Cos-7 cells have negligible ST3Gal-4 expression and were transduced with a ST3Gal-4 (OE) lentivirus. The ST3Gal-4 OE Cos-7 line showed increased ST3Gal-4 protein expression relative to EV and WT controls (Fig. S4, *A* and *B*). The total and activated EGFR protein levels were not significantly different between ST3Gal-4 OE, EV, and WT cells (Fig. S4, *A*, *C*, and *D*). We plated ST3Gal-4 OE and EV control cells on 56 pN TGT surfaces with and without EGF treatment. Epidermal growth factor stimulation led to increased spreading in both cell lines, but ST3Gal-4 OE was indistinguishable from the EV control (Fig. S4, *E* and *F*). Additionally, ST3Gal-4 OE did not alter the cell mechanics as measured by integrin tension (Fig. S4, *E* and *G*). Focal adhesion number or maturity were not impacted by ST3Gal-4 OE (Fig. S4, *E*, *H* and *I*). Unlike ST6Gal-I OE, ST3Gal-4 OE did not alter the EGFR activation or cell mechanical phenotypes.

**Figure 4:**
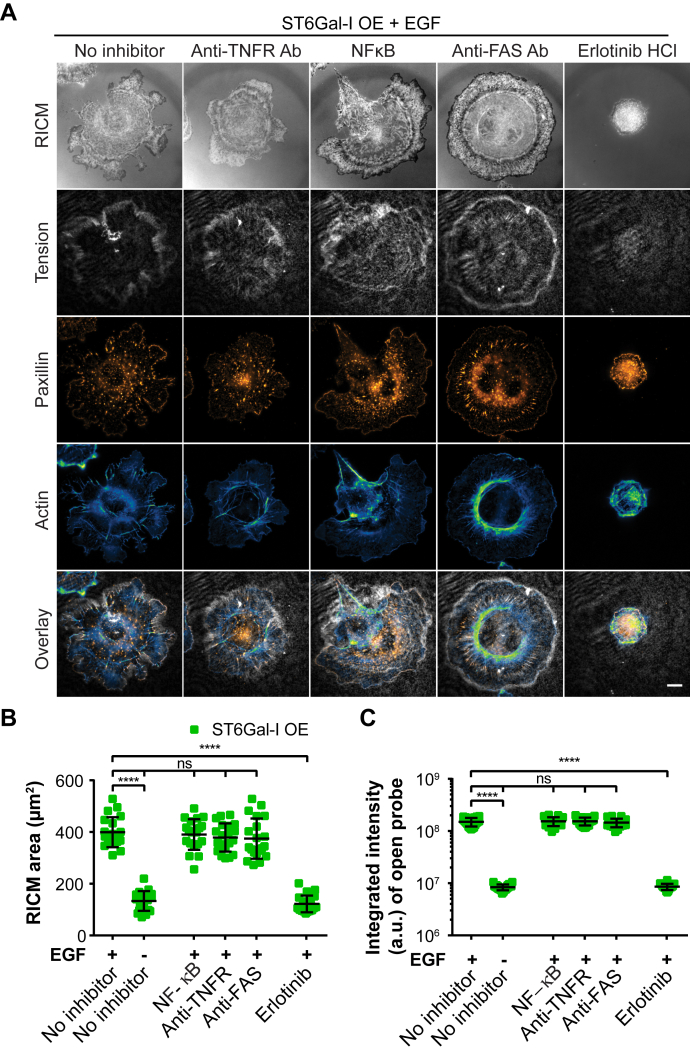
**ST6Gal-I sialylation of EGFR and not other cell surface receptors regulates cell spreading and integrin tension.***A*, ST6Gal-I overexpressing (OE) Cos-7 cells treated with control (DMSO – no inhibitor), NFκB inhibitor, anti-TNFR antibody, anti-FAS antibody, or erlotinib HCl in the presence or absence of EGF on a 56 pN TGT surface fixed and stained 90 min postplating. Shown here are RICM, integrin tension, paxillin, and actin, scale bar 10 μm. *B* and *C*, scatter plots for each treatment group with or without EGF stimulation of the (*B*) cell spread area (with EGF: No inhibitor, 399.3 ± 58.1 μm^2^, NFκB inhibitor, 390.4 ± 59.2 μm^2^, anti-TNFR antibody, 378.7 ± 54.7 μm^2^, anti-FAS antibody, 374.5 ± 77.8 μm^2^, erlotinib HCl, 122.1 ± 32.3 μm^2^; without EGF: no inhibitor, 133.4 ± 38.1 μm^2^) and (*C*) the integrated intensity of open probes (with EGF: No inhibitor, 1.7∗10^8^ ± 3.1∗10^7^ a.u., NFκB inhibitor, 1.7∗10^8^ ± 3.2∗10^7^ a.u., anti-TNFR antibody, 1.7∗10^8^ ± 3.2∗10^7^ a.u., anti-FAS antibody, 1.6∗10^8^ ± 3.3∗10^7^ a.u., erlotinib HCl, 8.8∗10^6^ ± 3.3∗10^6^ a.u.; without EGF: no inhibitor, 8.5∗10^6^ ± 3.1∗10^6^ a.u.). (mean ± SD, n = 25 cells across three independent sets of experiments; ^ns^p > 0.05, ∗∗∗∗*p* < 0.0001 by one-way ANOVA with Tukey’s test).

Next, we explored if EGFR or other proteins known to be sialylated by ST6Gal-I, such as tumor necrosis factor receptor (TNFR) and Fas cell surface death receptor (FasR), contributed to the observed mechanical phenotypes. ST6Gal-I OE cells were treated with control (DMSO – no inhibitor), anti-FAS antibody, anti-TNFR antibody, NFκB inhibitor, or erlotinib HCl (EGFR inhibitor) in the presence or absence of EGF on a 56 pN TGT surface (Fig. 4*A*). These proteins were selected based on the established influence of ST6Gal-I OE on their function and downstream signaling (29, 61, 62). There was no change in the spread area or integrated intensity of open probes in cells treated with anti-TNFR Ab, NFkB inhibitor, or anti-Fas Ab indicating these proteins do not play a role in the observed mechanical phenotypes. In contrast, treatment with erlotinib HCl reduced cell spreading and the integrated intensity of open probes to the same level as control cells with no EGF. This suggests that α2,6 sialylation of EGFR regulates the observed EGF-mediated changes in spread area and integrin tension (Fig. 4, *B* and *C*).

### ST6Gal-I–mediated EGFR sialylation promotes cell migration and invasion in an EGF-dependent manner

**Figure 5:**
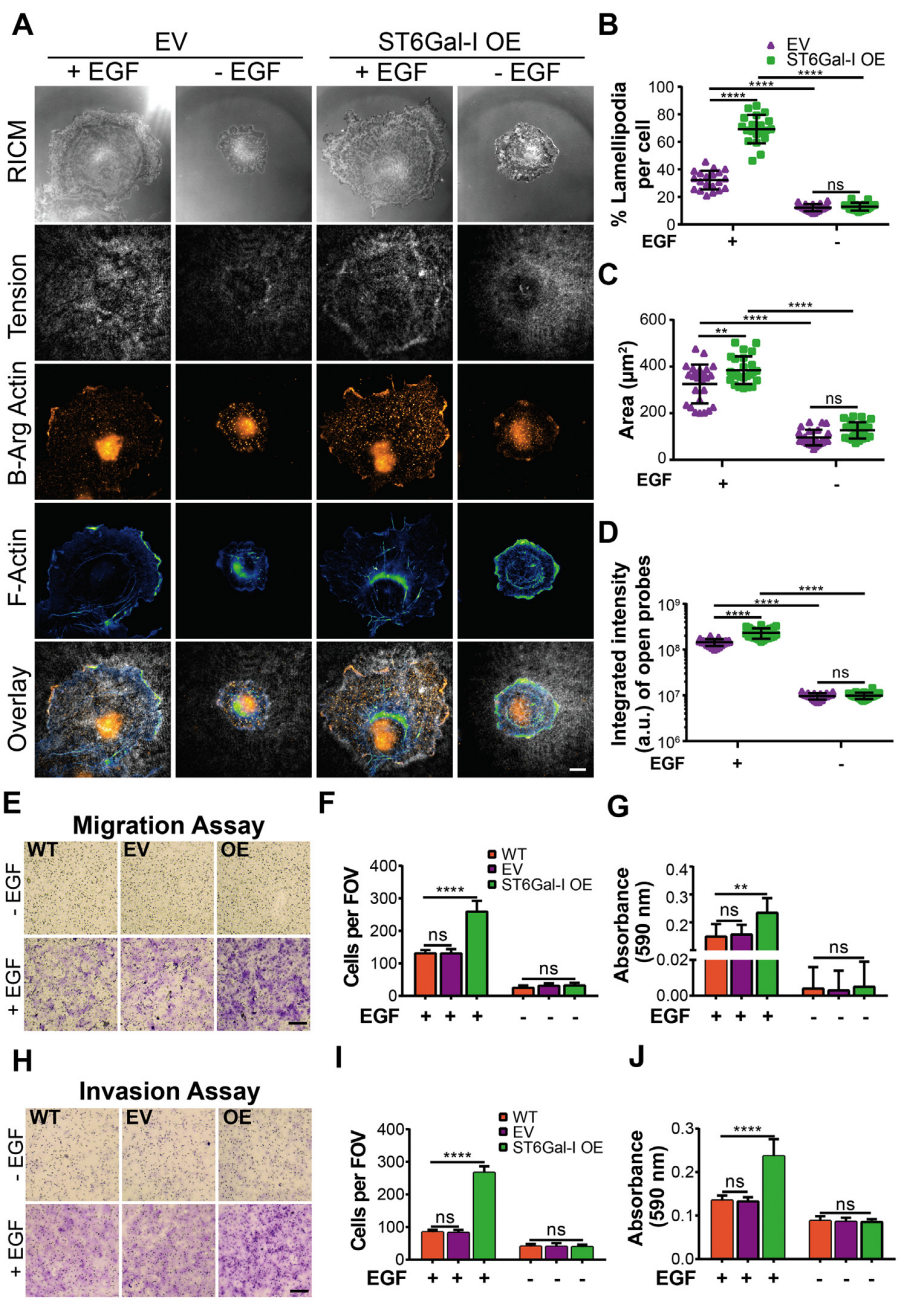
**ST6Gal-I expression promotes cell migration and invasion in an EGF-dependent manner.***A*, images of empty vector (EV) and ST6Gal-I overexpressing (OE) Cos-7 cells visualized by reflection interference contrast microscopy (RICM) and immunofluorescence, fixed, and stained 90 min postplating with or without EGF stimulation on a 56 pN TGT surface. Filamentous actin was stained with Alexa Fluor 488 phalloidin and β-actin with arginylated anti-beta actin antibody, scale bar 10 μm. *B*–*D*, scatter plots for (*B*) percentage lamellipodia per cell (EV, OE cells; with EGF: 32.2 ± 6.8%, 69.2 ± 10.3%; without EGF: 12.2 ± 2.6%, 12.9 ± 2.8%), (*C*) the cell footprint, RICM area (EV, OE cells; with EGF: 325.3 ± 83.3 μm^2^, 384.4 ± 59.3 μm^2^; without EGF: 95.7 ± 33.3 μm^2^, 126.9 ± 35.1 μm^2^), and (*D*) the integrated intensity of open probes (EV, OE cells with EGF: 1.5∗10^8^ ± 4.3∗10^7^ a.u., 3.6∗10^8^ ± 5.6∗10^7^ a.u.; without EGF: 1.0∗10^7^ ± 1.8∗10^6^ a.u., 1.1∗10^7^ ± 2.1∗10^6^ a.u.) for all cells within each group. (mean ± SD; n = 25 cells across three independent set of experiments; ^ns^*p* > 0.05, ∗∗*p* < 0.01, ∗∗∗∗*p* < 0.0001 by one-way ANOVA with Tukey’s test). *E*, images for transwell migration assays performed with WT, EV, and ST6Gal-I OE Cos-7 cells in the presence or absence of EGF stimulation, scale bar 250 μm, magnification, 10×. *F*, the migration ability calculated by counting cells per field of view (FOV) following crystal violet staining 24 h post plating (WT, EV, OE cells with EGF: 131.0 ± 9.7, 130.5 ± 13.4, 259.0 ± 33.4; without EGF: 24.7 ± 7.5, 31.2 ± 7.2, 32.0 ± 8.2). *G*, quantitative analysis of stained migratory Cos-7 cells performed on a microplate reader by recording absorbance at 590 nm (WT, EV, OE cells with EGF: 0.15 ± 0.05, 0.16 ± 0.040.23 ± 0.05; without EGF: 0.004 ± 0.01, 0.003 ± 0.01, 0.005 ± 0.01). *H*, images of the invasion assay performed on matrigel-coated transwells with WT, EV, and ST6Gal-I OE Cos-7 cells in the presence or absence of EGF stimulation, scale bar 250 μm, magnification, 10×. *I*, the invasion ability was estimated by counting cells per field of view following crystal violet staining 36 h post plating (WT, EV, OE cells with EGF: 84.2 ± 7.3, 82.3 ± 8.8, 266.5 ± 20.1; without EGF: 40.2 ± 8.1, 39.8 ± 11.3, 38.8 ± 7.5). *J*, quantitative analysis of crystal violet stained cells was performed on a microplate reader by recording absorbance at 590 nm (WT, EV, OE cells with EGF: 0.14 ± 0.01, 0.13 ± 0.01, 0.24 ± 0.04; without EGF: 0.09 ± 0.01, 0.09 ± 0.01, 0.09 ± 0.01; mean ± SD, n = 9 wells, across three sets of experiments, ^ns^p > 0.05, ∗∗*p* < 0.01, ∗∗∗∗*p* < 0.0001 by one-way ANOVA with Tukey’s test).

Previously, ST6Gal-I upregulation has been shown to induce a migratory and invasive phenotype in gastric, colon, liver, prostate, ovarian, pancreatic, breast, and cervical cancers (26, 30, 63, 64). Our results suggest ST6Gal-I influences FA turnover and maturation as indicated by higher number of FAs with smaller size on the 56 pN TGT surface (Fig. 2, *F* and *G*). Interestingly, we also observed that the morphology of ST6Gal-I OE cells was more variable than EV controls (Figs. 2*C* and S2*A*). Together, this suggested that ST6Gal-I might be promoting cell migration. To investigate this, we first asked if ST6Gal-I OE cells had enhanced lamellipodia identified by arginylated β-actin staining (Figs. 5*A* and S5). On the 56 pN TGT surface, ST6Gal-I OE cells had a higher percentage of lamellipodia compared to EV cells (Figs. 5*B* and S5). This increase in the migratory phenotype could partially explain the underlying mechanism driving smaller FAs observed on the 56 pN TGT surface (Fig. 2*G*). These cells also had larger cell spread area and higher integrated integrin tension (Fig. 5, *C* and *D*) as shown in Figure 2. To see if the increased formation of lamellipodia was driving motility, we next conducted a transwell migration assay. We found ST6Gal-I OE cells were significantly more migratory compared to EV controls when stimulated with EGF (Fig. 5, *E*–*G*). Furthermore, in an invasion assay ST6Gal-I OE cells show enhanced invasion across Matrigel-coated transwells when compared to EV cells with EGF treatment (Fig. 5, *H*–*J*). Together, these results support a relationship between EGFR α2,6 sialylation and the acquisition of pro-oncogenic migratory and invasive phenotypes (65, 66, 67).

### ST6Gal-I–mediated EGFR sialylation promotes cell proliferation and survival upon EGF stimulation

**Figure 6:**
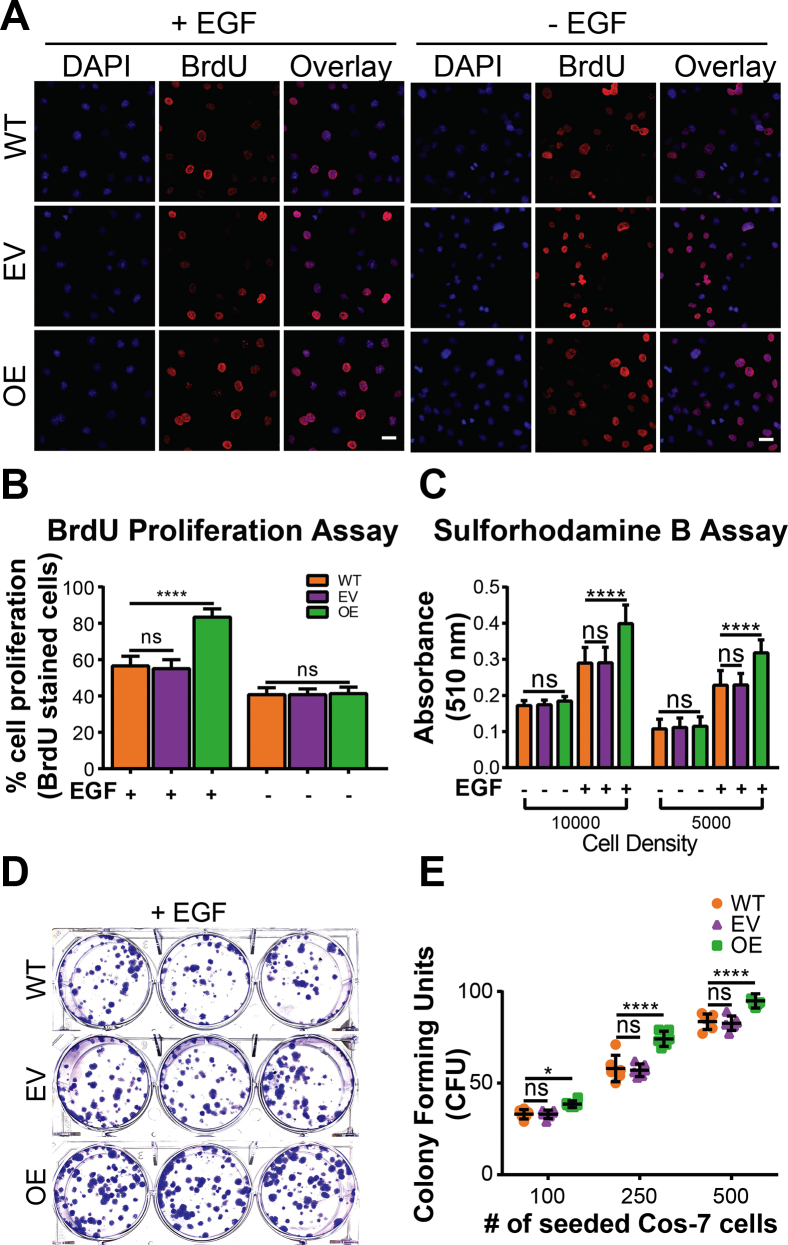
**ST6Gal-I promotes Cos-7 cell proliferation and survival.***A*, fluorescence images of the BrdU assay 6 h after treatment of WT, empty vector (EV), and ST6Gal-I overexpressing (OE) Cos-7 cells in the presence or absence of EGF stimulation. Cells are labeled with DAPI (nuclei, *blue*) and anti-BrdU Alexa Fluor 488 antibody (*red*). The scale bar represents 25 μm, magnification, 20×. *B*, cell proliferation was quantified by counting the percentage of BrdU positive cells across 10 fields of view. (WT, EV, OE cells with EGF: 56.5 ± 5.4%, 55.0 ± 5.0%, 83.3 ± 4.6%; without EGF: 40.7 ± 3.9%, 40.7 ± 3.2%, 41.3 ± 3.5%; n = 30 fields, across three independent experiments, ^ns^p > 0.05, ∗∗∗∗*p* < 0.0001 by one-way ANOVA with Tukey’s test). *C*, Sulforhodamine B (SRB) assay to quantitate proliferation by recording absorbance at 510 nm. (WT, EV, OE cells @10,000 cell density with EGF: 0.29 ± 0.04, 0.29 ± 0.04, 0.40 ± 0.05; without EGF: 0.17 ± 0.01, 0.17 ± 0.01, 0.18 ± 0.01; @ 5000 cell density with EGF: 0.23 ± 0.04, 0.23 ± 0.03, 0.32 ± 0.04; without EGF: 0.11 ± 0.03, 0.11 ± 0.03, 0.11 ± 0.03). *D*, images of the clonogenic assay for cell survival performed in 6-well plates for WT, EV, and ST6Gal-I OE Cos-7 cells with EGF (seeding density 100, 250, and 500 *left* to *right*). *E*, cell survival was evaluated by counting the number of colonies (CFU) formed 2 weeks postplating at different seeding densities. (WT, EV, OE cells with EGF: @100 seeding density: 33.0 ± 2, 33.0 ± 1.7, 38.5 ± 1.3; @250 seeding density: 58 ± 5, 57 ± 2.7, 74.2 ± 3.2; @500 seeding density: 83.5 ± 3.3, 82.7 ± 2.7, 94.8 ± 2.8; mean ± SD, n = 9, across three sets of experiments, ^ns^*p* > 0.05, ∗*p* < 0.05, ∗∗∗∗*p* < 0.0001 by one-way ANOVA with Tukey’s test).

We wanted to next explore proliferation and survival which could be influenced by enhanced EGFR activity. Epidermal growth factor receptor is a known regulator of cell proliferation, and sialylation can alter proliferative signaling cascades in different cancers both in the presence or absence of proliferative stimuli (40, 68, 69). We evaluated the effects of ST6Gal-I OE on Cos-7 cell proliferation with or without EGF stimulation. A BrdU assay showed that ST6Gal-I OE led to enhanced proliferation compared to EV and WT controls only in the presence of EGF stimulation (Fig. 6, *A* and *B*). This was validated to coincide with an increase in ST6Gal-I OE cell density by the colorimetric Sulforhodamine B assay, which quantitatively measures cellular protein content (Fig. 6*C*) (70). Activated EGFR facilitates cell survival (71, 72), and we next asked if ST6Gal-I OE enhanced cell survival though activated EGFR. To test this, we performed a clonogenic assay for ST6Gal-I OE, EV, and WT cells in the presence of EGF (Fig. 6*D*). ST6Gal-I OE cells had a significantly higher number of surviving colonies compared to EV or WT controls (Fig. 6*E*). This suggests that ST6Gal-I OE confers prosurvival characteristics to cells through activated EGFR.

### ST6Gal-I–mediated EGFR sialylation leads to sustained ERK and Akt activation

**Figure 7:**
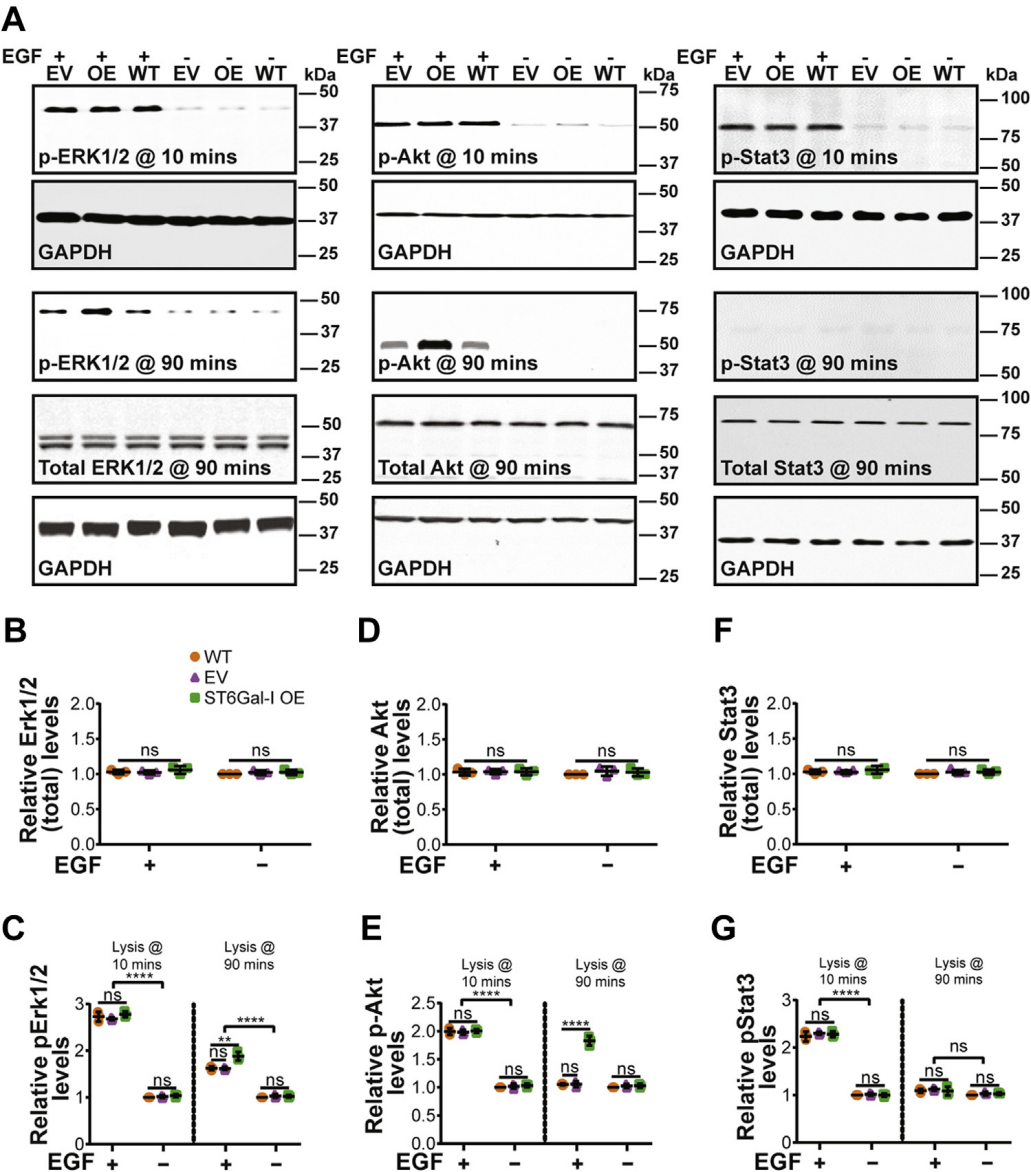
**ST6Gal-I leads to sustained Erk and Akt signaling.***A*, representative immunoblots for p-Erk1/2 (p44/42 MAPK), total Erk/MAPK, p-Akt, total Akt, p-STAT3, total STAT3, and GAPDH loading control for WT, EV, or ST6Gal-I OE Cos-7 cells with or without EGF treatment at 10- and 90-min post-stimulation. *B*–*G*, quantitative analysis of three independent blots for (*B*) total Erk/MAPK, (*C*) pErk1/2, (*D*) total Akt, (*E*) pAKt, (*F*) total Stat3, and (*G*) pStat3 in WT, EV, and ST6Gal-I OE Cos-7 cells (normalized to WT cells without EGF treatment). The groups were assessed statistically by ANOVA with Tukey’s test (mean ± SD, n = 3 independent sets of experiments; ^ns^*p* > 0.05, ∗∗∗∗*p* < 0.0001).

Since ST6Gal-I OE increases EGFR activity, we wanted to further dissect the roles of the downstream signaling pathways ERK, PI3K-Akt-mTOR, and JAK-STAT in the regulation of cell spreading, mechanics, migration, invasion, proliferation, and survival. ST6Gal-I OE did not affect the total ERK, Akt, or STAT protein expression levels measured by Western blotting compared to EV or WT controls (Fig. 7, *A*, *B*, *D*, and *F*). Epidermal growth factor stimulation led to increased activation of the signaling pathway proteins compared to nonstimulated (no EGF) controls at 10 min post-stimulation. At this time, the levels of activated ERK, Akt, and STAT were not significantly different between ST6Gal-I OE, EV, and WT cells (Fig. 7, *A*, *C*, *E*, and *G*). However, at 90 min post-EGF stimulation, the ERK and Akt pathways showed sustained activation with ST6Gal-I OE compared to controls while STAT returned to baseline levels irrespective of EGF stimulation.

### ST6Gal-I sialylation promotes cell migration *via* sustained Akt activation

**Figure 8:**
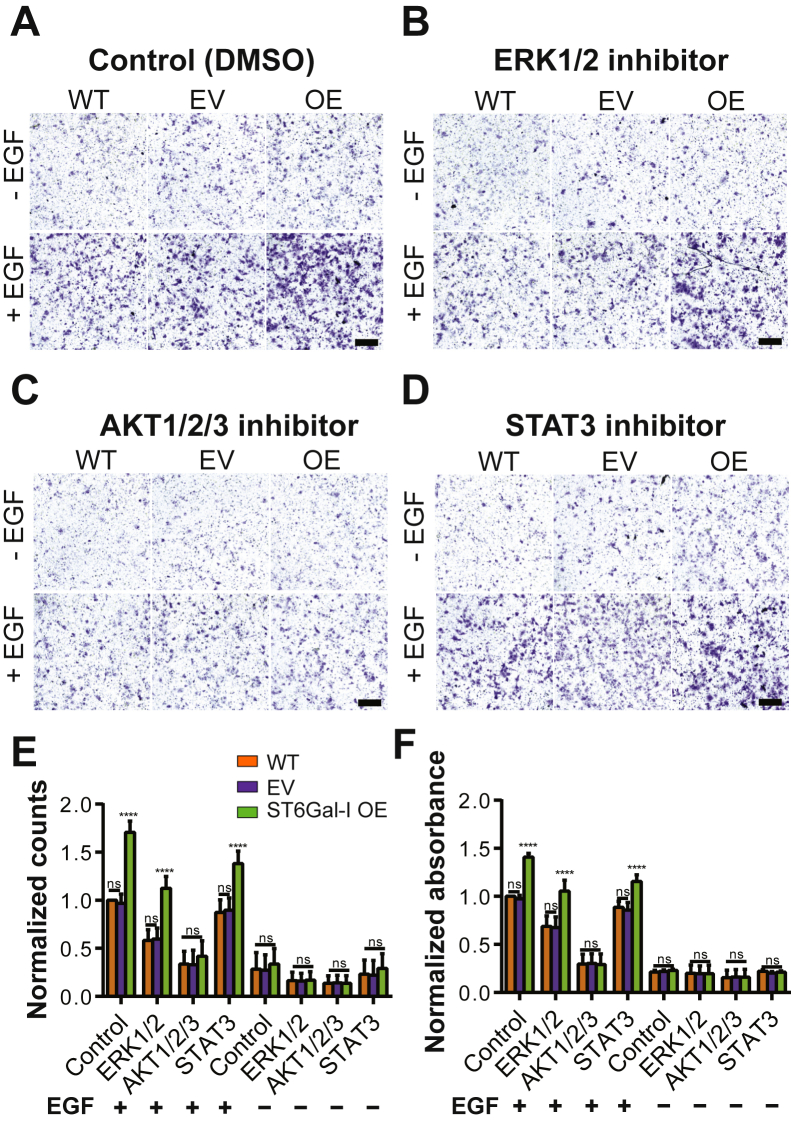
**ST6Gal-I promotes Cos-7 cell migration in an AKT-dependent manner.***A*–*D*, images for transwell migration assays performed with specific pathway inhibitors for WT, empty vector (EV), and ST6Gal-I overexpressing (OE) Cos-7 cells in the presence or absence of EGF, scale bar 250 μm, magnification, 10×. Representative images showing crystal violet-stained cells treated with (*A*) vehicle (DMSO- control), (*B*) ERK inhibitor, (*C*) AKT inhibitor, and (*D*) STAT inhibitor with or without EGF stimulation. *E*, the migratory ability of cells evaluated by counting cells per field of view (FOV) 24 h post-plating and normalized to WT control with EGF (WT, EV, ST6Gal-I OE cells; with EGF: Ctrl, 1.00 ± 0.00, 0.97 ± 0.10, 1.70 ± 0.12; ERK, 0.58 ± 0.11, 0.59 ± 0.12, 1.12 ± 0.12; AKT, 0.34 ± 0.13, 0.33 ± 0.15, 0.42 ± 0.16; STAT, 0.87 ± 0.13, 0.90 ± 0.13, 1.38 ± 0.13. without EGF: Ctrl, 0.28 ± 0.17, 0.27 ± 0.16, 0.34 ± 0.16; ERK, 0.16 ± 0.08, 0.16 ± 0.08, 0.17 ± 0.09; AKT, 0.14 ± 0.08, 0.14 ± 0.08, 0.14 ± 0.08; STAT, 0.23 ± 0.15, 0.22 ± 0.15, 0.29 ± 0.15). *F*, quantitative analysis was performed on a microplate reader by recording absorbance at 590 nm and normalized to WT control with EGF (WT, EV, ST6Gal-I OE cells; with EGF: Ctrl, 1.00 ± 0.00, 0.97 ± 0.04, 1.41 ± 0.04; ERK, 0.69 ± 0.11, 0.68 ± 0.11, 1.05 ± 0.11; AKT, 0.30 ± 0.10, 0.30 ± 0.10, 0.29 ± 0.11; STAT, 0.89 ± 0.06, 0.86 ± 0.08, 1.15 ± 0.07; without EGF: Ctrl, 0.21 ± 0.02, 0.22 ± 0.02, Ctrl, 0.23 ± 0.01, ERK, 0.20 ± 0.09, 0.20 ± 0.08, 0.20 ± 0.09; AKT, 0.15 ± 0.08, 0.16 ± 0.08, 0.16 ± 0.08; STAT, 0.22 ± 0.02, 0.20 ± 0.01, 0.21 ± 0.01). (mean ± SD, n = 9 wells, across three sets of experiments, ^ns^p > 0.05, ∗∗∗∗*p* < 0.0001 by one-way ANOVA with Tukey’s test).

Next, we wanted to tease apart the distinct roles for the ERK, PI3K-Akt-mTOR, or JAK-STAT pathways downstream of activated EGFR in promoting cell migration. Inhibition of the ERK1/2 (SCH772984) and STAT3 (Niclosamide) pathways led to a decrease in migration in all cells, and the differences between the ST6Gal-I OE and EV and WT controls were conserved (Fig. 8, *A*–*F*). In contrast, inhibition of the AKT1/2/3 pathway (MK-2206 dihydrochloride) led to decreased migration in all cells and the ST6Gal-I OE cells were not significantly different than EV or WT cells. This suggests that the Akt pathway activation regulates EGF stimulation–dependent cell migration in ST6Gal-I OE cells.

### ST6Gal-I sialylation promotes cell tension *via* sustained ERK activation

**Figure 9:**
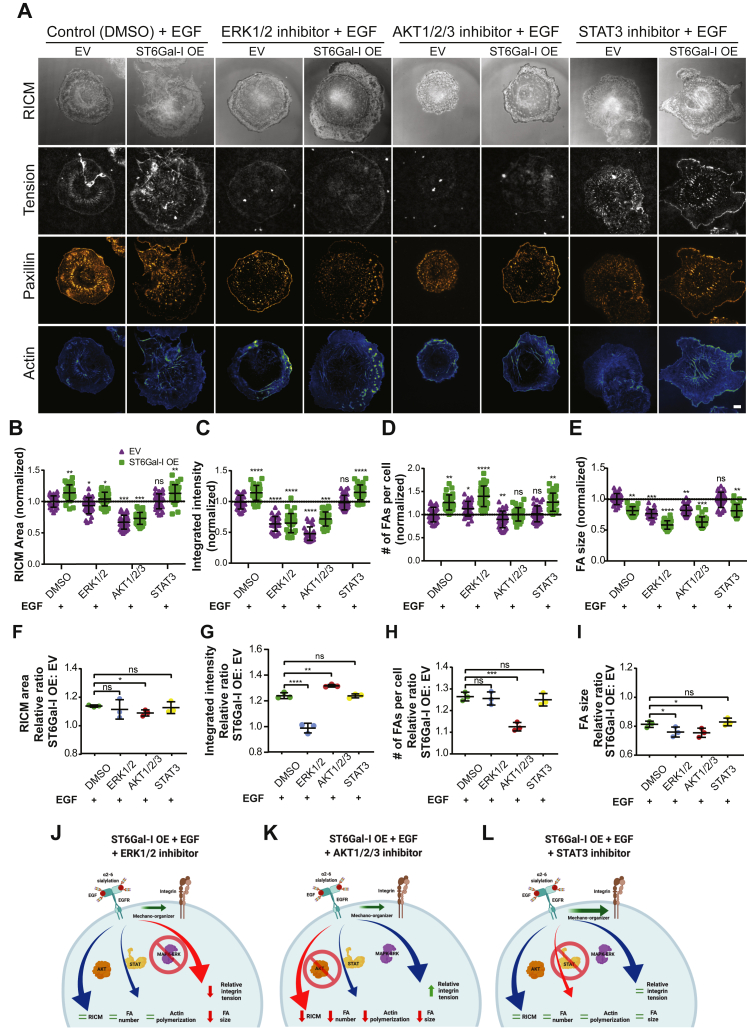
**ST6Gal-I promotes mechanical tension outcome primarily *via* ERK signaling.***A*, images for control (vehicle, DMSO) and inhibitor (ERK, AKT, and STAT) treated empty vector (EV) and ST6Gal-I overexpressing (OE) Cos-7 cells with EGF on the 56 pN TGT surface 90 min post-plating. The images include RICM, integrin tension (*gray*), paxillin (*orange hot*), and actin (*blue-green*), scale bar 10 μm. *B*–*E*, scatter plots normalized to control treated EV cells for the (*B*) RICM area (EV, OE cells: DMSO, 1.00 ± 0.09, 1.14 ± 0.13; ERK, 0.93 ± 0.13, 1.04 ± 0.11; AKT, 0.67 ± 0.11, 0.73 ± 0.09; STAT, 1.01 ± 0.11, 1.13 ± 0.14), (*C*) integrated intensity of open probes (EV, OE cells: DMSO, 1.00 ± 0.11, 1.14 ± 0.12; ERK, 0.64 ± 0.12, 0.65 ± 0.16; AKT, 0.48 ± 0.11, 0.72 ± 0.11; STAT, 0.98 ± 0.12, 1.15 ± 0.12), (*D*) number of focal adhesions (FAs) per cell (EV, OE cells: DMSO, 1.00 ± 0.16, 1.26 ± 0.16; ERK, 1.13 ± 0.16, 1.40 ± 0.22; AKT, 0.90 ± 0.18, 1.01 ± 0.14; STAT, 1.02 ± 0.17, 1.27 ± 0.20), and (*E*) focal adhesion (FA) size (EV, OE cells: DMSO, 1.00 ± 0.09, 0.81 ± 0.06; ERK, 0.76 ± 0.07, 0.58 ± 0.07; AKT, 0.82 ± 0.08, 0.63 ± 0.08; STAT, 0.99 ± 0.12, 0.81 ± 0.10). *F*–*I*, cumulative plots showing the relative ST6Gal-I OE to EV ratios for the morphometric and mechanical outcomes for each experimental set measured above: (*F*) RICM spread area (DMSO, 1.14 ± 0.01; ERK, 1.11 ± 0.07; AKT, 1.09 ± 0.02; STAT, 1.13 ± 0.04), (*G*) integrated intensity of open probes (DMSO, 1.22 ± 0.02; ERK, 0.98 ± 0.01; AKT, 1.33 ± 0.06; STAT, 1.24 ± 0.04), (*H*) number of focal adhesions (FAs) per cell (DMSO, 1.26 ± 0.02; ERK, 1.26 ± 0.03; AKT, 1.13 ± 0.02; STAT, 1.25 ± 0.02), and (*I*) focal adhesion (FA) size (DMSO, 0.81 ± 0.02; ERK, 0.76 ± 0.03; AKT, 0.75 ± 0.03; STAT, 0.83 ± 0.03). *J*–*L*, schematic representation of the changes in cell morphology and mechanical outcomes as a consequence of (*J*) ERK, (*K*) AKT, and (*L*) STAT inhibitor treatment. (mean ± SD, n = 25 cells across three independent experiments; ^ns^*p* > 0.05, ∗*p* < 0.05, ∗∗*p* < 0.01, ∗∗∗*p* < 0.001, ∗∗∗∗*p* < 0.0001 by one-way ANOVA with Tukey’s test).

We also wanted to identify the roles of the signaling cascades downstream of EGFR in cell spreading, FA formation, and integrin tension generation. ST6Gal-I OE and EV Cos-7 cells were plated on 56 pN TGT surfaces in the presence of EGF and treated with either DMSO (control) or inhibitors for ERK (SCH772984), Akt (MK-2206 dihydrochloride), or JAK-STAT (Niclosamide) (Fig. 9*A*). ERK inhibition led to a decrease in cell spread area compared to DMSO-treated controls (Fig. 9*B*). However, the ratio of the spread area (ST6Gal-I OE/EV) was maintained, indicating that ERK inhibition affected both OE and EV cells similarly (Fig. 9*F*). ERK inhibition led to a significant decrease in total integrated tension (Fig. 9*C*) and the difference between the ST6Gal-I OE and EV cells was reduced, suggesting ST6Gal-I OE is driving increased integrin tension though the Erk pathway (Fig. 9*G*). In contrast, Akt inhibition reduced the area of both OE and EV cells. The relative ratio of cell area was significantly lower when compared to DMSO-treated controls, indicating Akt is involved in cell spreading (Fig. 9*F*). Additionally, there was an increase in the relative ratio of integrated intensity of open probes indicating a larger effect on ST6Gal-I OE cells compared to EV cells (Fig. 9*G*). Additionally, there was an increase in the relative ratio of integrated intensity of open probes indicating a disproportionate effect on ST6Gal-I OE and EV cells. Akt has a role in regulating the integrin tension generation, but this role is not enhanced with ST6Gal-I expression. This suggests that the Akt pathway enhances cell spreading and integrin tension in ST6Gal-I OE cells while the ERK pathway regulates integrin tension. We found ERK inhibition led to an increased number of FAs, while the size of those FAs decreased, possibly suggesting stalled maturation (Fig. 9, *D*, *E*, *H*, and *I*). AKT inhibition significantly reduced the relative number of FAs in OE and EV cells.

Inhibition of the STAT pathway did not differentially affect ST6Gal-I OE cells compared to EV cells suggesting it does not regulate cell spreading or integrin tension (Fig. 9, *B*–*I*). Similar results for all inhibitors were observed on 12 pN TGT surfaces, suggesting that the tension threshold of the underlying substrate does not alter the roles of the ERK and AKT pathways in these processes (Fig. S6). Overall, we found the ERK signaling cascade is involved in integrin tension generation while the PI3K/AKT pathway primarily regulates cell spreading and FA maturation (Fig. 9*J*).

### ST6Gal-I sialylation promotes EGFR surface maintenance and thereby sustained signaling

**Figure 10:**
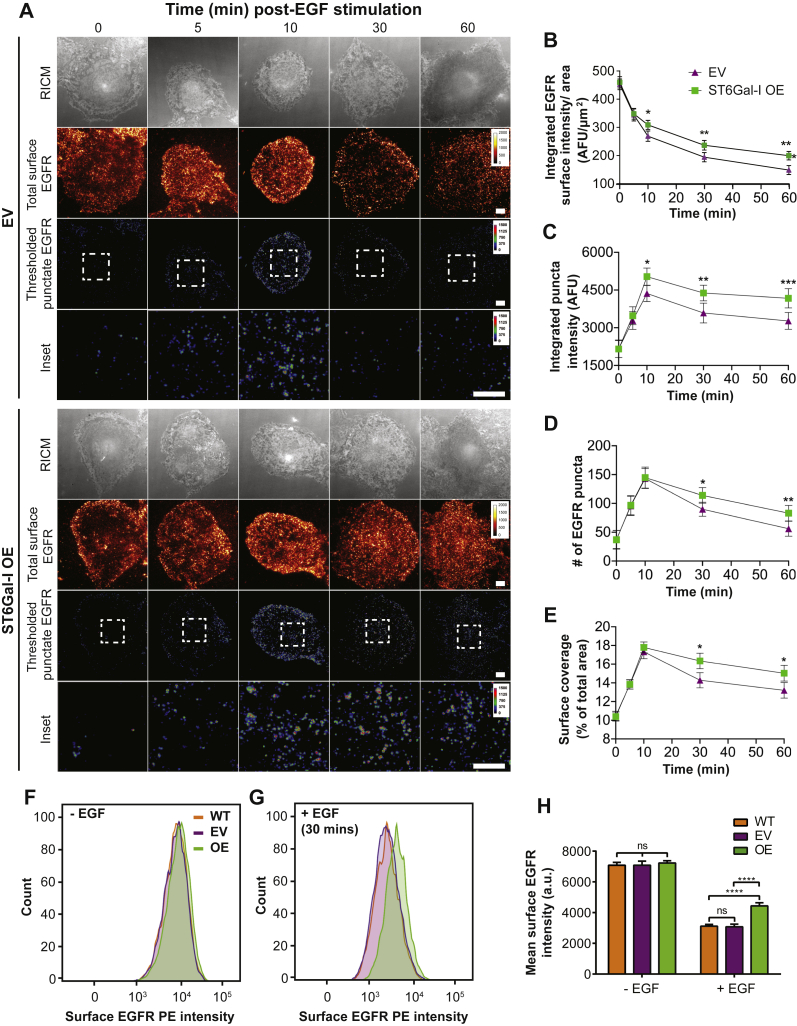
**ST6Gal-I–mediated EGFR sialylation promotes EGFR surface maintenance and sustained signaling.***A*, images for empty vector (EV) and ST6Gal-I overexpressing (OE) Cos-7 cells on a glass surface fixed and stained at different timepoints post-EGF stimulation visualized by reflection interference contrast microscopy (RICM) and total internal reflection fluorescence microscopy (TIRFM). The images depict the surface EGFR distribution and clustered EGFR (puncta) following thresholding (rainbow RGB), scale bar 10 μm. Insets highlight EGFR clusters (puncta), scale bar 3 μm. *B*–*E*, quantification of the (*B*) integrated surface EGFR intensity normalized to area (0, 5, 10, 30, and 60 min for EV: 449.3 ± 19.7 a.u./μm^2^, 348.6 ± 16.8 a.u./μm^2^, 279.2 ± 18.8 a.u./μm^2^, 201.1 ± 18.5 a.u./μm^2^, 148.7 ± 18.7 a.u./μm^2^; OE: 457.6 ± 19.2 a.u./μm^2^, 351.3 ± 17.1 a.u./μm^2^, 311.8 ± 16.9 a.u./μm^2^, 249.6 ± 17.2 a.u./μm^2^, 215.3 ± 16.4 a.u./μm^2^), (*C*) average integrated intensity for EGFR puncta identified by thresholding (0, 5, 10, 30, and 60 min EV: 2165.7 ± 350.4 a.u., 3287.8 ± 352.2 a.u., 4362.7 ± 320.8 a.u., 3587.8 ± 394.1 a.u., 3273.5 ± 334.1; OE: 2154.7 ± 339.1 a.u., 3479.8 ± 357.6 a.u., 5033.5 ± 342.5 a.u., 4383.2 ± 312.6 a.u., 4172.2 ± 382.3 a.u.), (*D*) average number of EGFR puncta per cell (0, 5, 10, 30, and 60 min EV: 37.4 ± 15.8, 95.8 ± 16.7, 143.3 ± 17.4, 89.9 ± 12.3, 56.1 ± 13.1; OE: 36.9 ± 16.2, 96.7 ± 16.6, 144.8 ± 18.3, 113.8 ± 13.5, 83.1 ± 13.5), and (*E*) percent of area containing EFGR puncta (0, 5, 10, 30, and 60 min EV: 10.5 ± 2.5%, 13.8 ± 2.5%, 17.3 ± 3.7%, 14.3 ± 4.1%, 13.2 ± 4.1%; OE: 10.4 ± 2.4%, 13.9 ± 2.5%, 17.8 ± 2.9%, 16.3 ± 4.2%, 15.0 ± 4.2%) following EGF stimulation. (mean ± SD, n = 30 cells across three independent experiments; ^ns^p > 0.05, ∗*p* < 0.05, ∗∗*p* < 0.01, ∗∗∗*p* < 0.001, ∗∗∗∗*p* < 0.0001 by ANOVA with Tukey’s test). *F* and *G*, surface EGFR staining to verify EGFR surface levels in WT, EV, and ST6Gal-I OE cells as detected by flow cytometry (*F*) without or (*G*) with EGF (30 min) stimulation. *H*, quantification of the mean surface EGFR intensity (WT, EV, OE cells without EGF: 7084.3 ± 183.8, 7086.3 ± 266.2, 7230.0 ± 148.0; with EGF: 3119.7 ± 108.9, 3078.0 ± 174.7, 4435.3 ± 210.9; mean ± SD, across three independent experiments; ^ns^p > 0.05, ∗∗∗∗*p* < 0.0001 by ANOVA with Tukey’s test).

Finally, we investigated the mechanism behind the ST6Gal-I determined increase in EGFR activity. We hypothesized this was driven by increased localization of EGFR at the plasma membrane. To test this, we imaged the surface distribution of EGFR in ST6Gal-I OE and EV cells at different times following EGF stimulation. Only the surface pool of EGFR was labeled, and the cells were imaged using TIRF microscopy, which selectively excites fluorophores at or near the basal membrane (Fig. 10*A*). We first analyzed the total intensity, which represents the total EGFR localized at the plasma membrane (Fig. 10, *A* and *B*). The surface protein level was similar in ST6Gal-I OE and EV cells at time 0 when normalized to the cell area (Fig. 10*B*). This suggests that ST6Gal-I OE does not change the relative amount of EGFR at the plasma membrane. Following EGF stimulation, the amount of surface EGFR decreased in both cell lines, corresponding with receptor internalization. However, there was significantly more EGFR at the plasma membrane in ST6Gal-I OE cells from 10 min post-EGF stimulation. This suggests that ST6Gal-I OE decreased the rate of EGFR clearance from the plasma membrane. Next, we analyzed the distribution of EGFR into clusters, or puncta, which can represent accumulation for internalization or a signaling hub. In both cell lines, there was an initial increase in EGFR fluorescence from puncta in the first 10 min following stimulation (Fig. 10*C*). Between 10 and 30 min, the number of puncta decreased in both cell lines, as EGFR is internalized. However, the rate of decrease was lower in the ST6Gal-I OE cells. The percent of the membrane area occupied by EGFR puncta and the intensity of these puncta followed similar dynamics (Fig. 10, *C*–*E*). Epidermal growth factor receptor plasma membrane localization was confirmed by flow cytometry. ST6Gal-I OE, WT, or EV Cos-7 cells were fixed 30 min after EGF or mock stimulation and stained with phycoerythrin-conjugated anti-EGFR monoclonal antibody. ST6Gal-I OE cells showed enhanced surface persistence following 30 min of EGF stimulation, directly corresponding to the imaging results (Fig. 10, *F*–*H*). These results support a mechanism where ST6Gal-I OE leads to the maintenance of activated EGFR at the cell membrane.

### ST6Gal-I–mediated EGFR sialylation modulates cell mechanical outcomes across different cell lines

**Figure 11:**
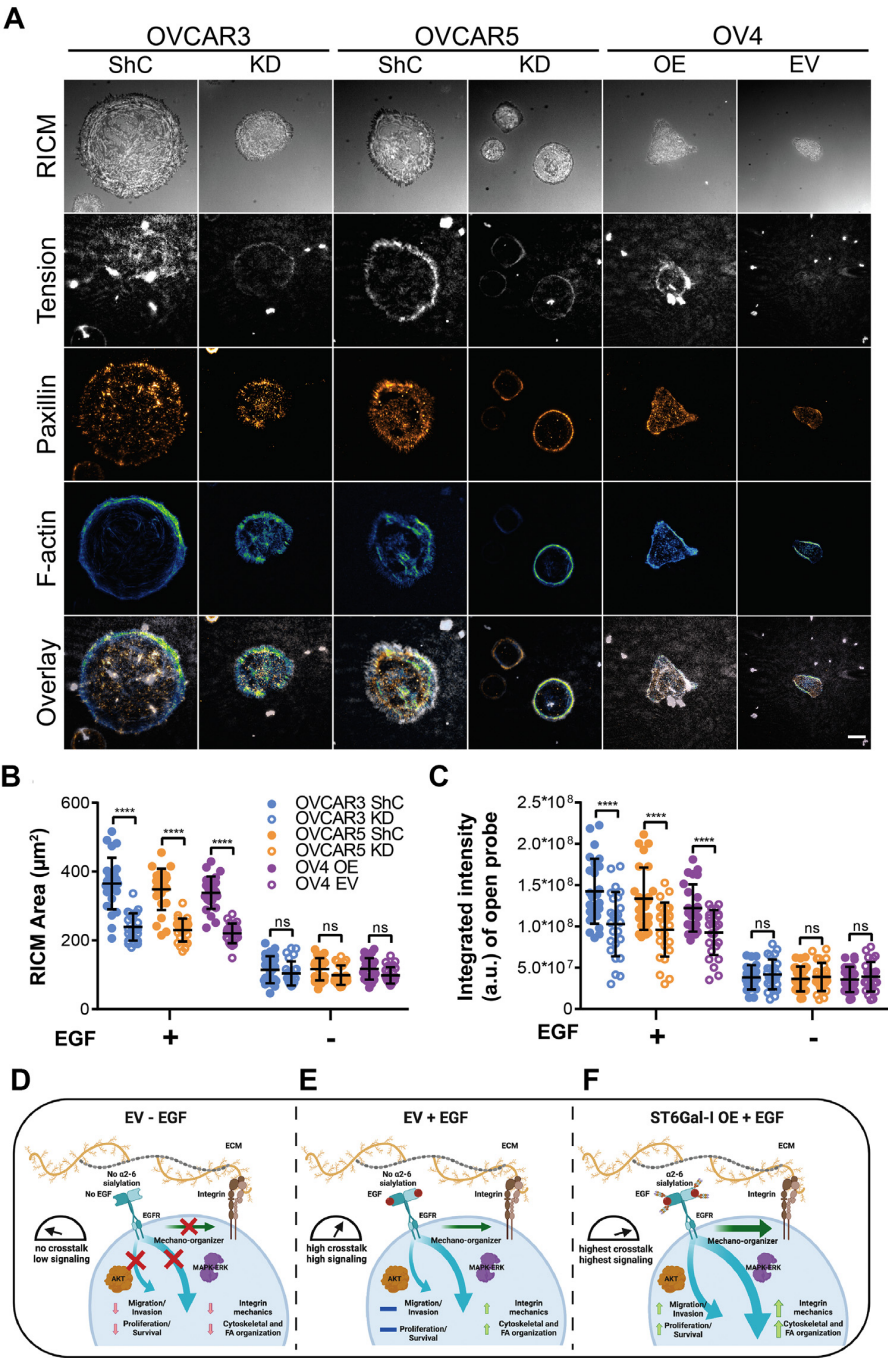
**ST6Gal-I–mediated EGFR sialylation is a molecular determinant of cell mechanics and invasion.***A*, images of OVCAR3 cells with a control shRNA (shC) or ST6Gal-I knockdown (KD) cells, OVCAR5 cells with shC or ST6Gal-I KD cells, and OV4 ST6Gal-I overexpressing (OE) and empty vector (EV) cells on a 56 pN TGT surface 90 min postplating in the presence of EGF (RICM, integrin tension - *grayscale*, paxillin - *orange hot*, and actin - *green blue*; scale bar 10 μm). *B* and *C*, quantification of the (*B*) cell spread area (OVCAR3 ShC, OVCAR3 KD, OVCAR5 ShC, OVCAR5 KD, OV4 OE, and OV4 EV cells with EGF: 365.2 ± 74.8, 239.0 ± 39.8, 348.4 ± 60.0, 229.8 ± 33.3, 338.4 ± 47.1, 220.4 ± 28.4 μm^2^; without EGF: 114.4 ± 39.2, 103.8 ± 34.8, 116.0 ± 32.04, 98.9 ± 28.1, 117.3 ± 31.1, 98.2 ± 24.1 μm^2^) and the (*C*) integrated intensity of open probes (OVCAR3 ShC, OVCAR3 KD, OVCAR5 ShC, OVCAR5 KD, OV4 OE, and OV4 EV cells with EGF: 1.4∗10^8^ ± 3.9∗10^7^, 1.0∗10^8^ ± 3.8∗10^7^, 1.3∗10^8^ ± 3.7∗10^7^, 9.6∗10^7^ ± 3.3∗10^7^, 1.2∗10^8^ ± 2.8∗10^7^, 9.3∗10^7^ ± 2.7∗10^7^ a.u.; without EGF: 3.8∗10^7^ ± 1.5∗10^7^, 4.2∗10^7^ ± 1.8∗10^7^, 3.6∗10^7^ ± 1.5∗10^7^, 3.8∗10^7^ ± 1.7∗10^7^, 3.6∗10^7^ ± 1.5∗10^7^, 3.9∗10^7^ ± 1.8∗10^7^ a.u.) for the different cell lines with or without EGF stimulation (mean ± SD, n = 25 cells across three independent experiments; ^ns^*p* > 0.05, ∗∗∗∗*p* < 0.0001 by one-way ANOVA with Tukey’s test). *D*–*F*, model highlighting how ST6Gal-I modulates EGF-dependent cell spreading, integrin mechanotransduction, FAs, cell proliferation, invasion, and survival. *D*, in the absence of EGF stimulation, limited integrin engagement of extracellular matrix results in limited activation of Akt and ERK integrin-signaling pathways, cytoskeletal organization, and FA maturation. *E*, ligand-dependent EGFR signaling increases integrin mechanotransduction and enhances FA maturation and cytoskeletal organization, primarily driven by the ERK-signaling pathway and not the Akt-signaling pathway. In this way, EGFR crosstalk modulates integrin-based cell mechanics. *F*, ST6Gal-I expression enhances ligand-dependent EGFR crosstalk leading to a collaborative amplification by the ERK and Akt signaling pathways. ERK signaling enhances the EGFR driven cell mechanics and facilitates integrin tension generation by tuning the threshold for integrin tension. ERK signaling also facilitates cell spreading through cytoskeletal and FA reorganization. In contrast, the sustained Akt signaling drives increased cell migration, invasion, and survival.

To test whether these findings were generalizable and applicable to human cell lines, we generated a panel comprising multiple cancer lines: OVCAR3 (human epithelial ovarian adenocarcinoma), OVCAR5 (originally thought to represent a high-grade ovarian carcinoma line, but suspected to be a human epithelial metastatic gastrointestinal carcinoma (73)), and OV4 (human epithelial ovarian carcinoma). Ovarian cancer is one of the deadliest gynecological malignancies primarily because of late detection and acquired drug resistance (74, 75). ST6Gal-I levels are often increased in the advanced stages of ovarian cancer and correlated with high tumor grade, metastasis, and reduced patient prognosis (27, 76). OVCAR3 and OVCAR5 cells have robust ST6Gal-I expression while OV4 cells lack endogenous ST6Gal-I, providing a unique experimental cell panel. We stably-transduced OVCAR3 and OVCAR5 cells with ST6Gal-I silencing or control shRNA lentivirus to generate knockdown (KD) and expressing (shRNA control, ShC) lines. OV4 cells were transduced with ST6Gal-I or control EV lentivirus to generate OV4 OE and EV lines. ST6Gal-I expression and cell surface α2,6 sialylation were validated by Western blot and flow cytometry (Fig. S7). The cells were plated on the 56 pN TGT surfaces with or without EGF stimulation to investigate the cell mechanical outcomes (Fig. 11*A*). Consistent with our results in the ST6Gal-I OE Cos-7 cells, EGF stimulation led to enhanced cell spreading and integrin tension in the ST6Gal-I expressing lines (OVCAR3 ShC, OVCAR5 ShC, and OV4 OE) compared to controls (Fig. 11, *B* and *C*). These results demonstrate the generalizability of our findings and extend the mechanistic role of ST6Gal-I–mediated EGFR sialylation in regulating cell mechanics across different cancer cell lines.

## Discussion

Aberrant sialylation is increasingly recognized as a near universal feature of cancer cells, and ST6Gal-I–mediated sialylation is implicated in promoting cancer hallmarks and modulating pathways intrinsic to tumor cell biology (27, 28, 77). Epidermal growth factor receptor is frequently mutated and/or overexpressed across different cancers and is the primary target for diverse cancer treatment strategies currently adopted in clinical practice (78, 79). While prior research has explored the individual roles of ST6Gal-I and EGFR in cancer progression from genetic and biochemical perspectives, an understanding of how these proteins work together to impact cell mechanics remains unexplored. We recently demonstrated that activated EGFR enhances integrin mechanics, cell spreading, and FA organization and maturation (22). We proposed that EGFR acts as a mechano-organizer, where in coordination with integrins, it tunes a cell’s ability to generate tension by lowering the activation threshold for spreading and enhancing FA organization (22). Since ST6Gal-I OE elevates EGFR activation, in this work, we explored the impact of sialylation on cell mechanics and the effect of these mechanical changes as determinants of cell behavior and morphology. We found ST6Gal-I OE promoted cell spreading and FA maturation in an activated EGFR-dependent manner (Fig. 11, *D*–*F*). Using the TGT platform, which reports a cell’s force history, we found ST6Gal-I OE led to increased tension generation by integrins. Employing classical cancer biology assays, we verified that ST6Gal-I OE enhanced mechanosignaling-increased migration, invasion, proliferation, and survival (80, 81). Because cells spread more on both the low and high T_tol_ TGT probes, we suggest that EGFR sialylation by ST6Gal-I modulates the loading capabilities of integrins, independent of the tension threshold of the underlying substrate (82, 83). Additionally, we saw an increase in actin organization in ST6Gal-I OE cells. The increased EGFR activity could regulate the interaction of contractile transverse actin fibers containing myosin with radial actin filaments and thereby modulate transmission of forces to FAs *via* integrins (84, 85). Previously, elevated EGFR kinase activity has been directly associated with malignant transformations (86, 87, 88, 89). Here, using multiple ovarian cancer cell lines, we show that ST6Gal-I can amplify the mechanical phenotypes in cancer cells to promote cell behaviors that are associated with tumorigenic potential. We find that sialylation of EGFR fine tunes EGFR-integrin crosstalk driving cell adhesion, migration, invasion, and proliferation.

Our results support previous findings and provide insight into the mechanism of how α2,6 sialylation of EGFR drives pro-oncogenic phenotypes through changed biophysical properties (34, 90, 91). We hypothesize this evolution is driven in part by changes in cell mechanics, including increased integrin tension and lowered activation threshold. These mechanical changes alter the cell’s ability to sense and respond to microenvironmental cues and promote spreading, migration, and invasion (21, 43). The TGT platform provides mechanistic evidence in support of these claims and helps establish that α2,6 sialic acid addition dynamically tunes cell mechanics by enhancing integrin tension and FA turnover. The evolution of cancer, from the initial tumor through metastasis, involves changes in the cell’s ability to sense and respond to their microenvironment and its mechanical properties (41, 92, 93). Our results provide mechanistic evidence that ST6Gal-I can dynamically tune the cell adhesion and migration *via* activated EGFR signaling.

Sialylation is a molecular signature that has gained enormous attention because of its association with multiple cancers. Since ST6Gal-I–mediated EGFR sialylation led to increased EGFR activity, we were interested in which downstream signaling cascades regulate the increased cell mechanics and tumor-promoting cell behaviors. The ERK, PI3K-AKT, and JAK-STAT pathways have been established to play overlapping roles in cancer cell signaling and facilitate cell proliferation, migration, regulate metabolism, and inhibit apoptotic pathways (94). However, their roles in regulating cell mechanics remains under investigated. Using specific inhibitors against ERK, PI3K-AKT, and JAK-STAT signaling pathways, we were able to ascertain the specific downstream EGFR-signaling cascade regulating mechanical outcomes and discern it from the pathway regulating cell morphometric changes. While changes in cell mechanical properties including increased integrin tension, FA nucleation, and promotion of cell spread area were attributed to the ERK-signaling pathway, the increased cellular migration, invasion, proliferation, and survival were controlled *via* the AKT cascade (Fig. 9) (26, 63, 90, 91). Our observations uncover the previously unexplored role of ERK signaling in regulating cell mechanics and align with previous results that show ST6Gal-I enhances ERK- and AKT-dependent survival signaling and promote epithelial–mesenchymal transition and cell invasiveness (28, 30, 63, 95, 96).

N-glycan branching has been reported to significantly increase the surface stability of membrane glycoproteins, including EGFR, through a mechanism involving N-glycan binding to the galectin lattice (97). In murine mammary epithelial cancer cells, galectin-3 binding to β1,6-acetylglucosaminyltransferase V (MGAT5)-modified N-glycans restricted the mobility of EGFR in the plasma membrane (98). Since MGAT5 catalyzes β1,6-GlcNAc branching of N-glycans, remodeling of the N-glycan structure *via* altered MGAT5 expression could potentially influence EGFR surface persistence observed in our ST6Gal-I OE cells (99, 100). Additionally, growing evidence has shown that both ST6Gal-I and MGAT5 promote cell migration and invasion (101, 102). However, we did not observe any significant MGAT5 expression in our WT, EV, or ST6Gal-I OE Cos-7 cells (Fig. S8*A*). Blocking galectin-binding sites by pretreating cells with galactose did not alter the ability of cells to bind, spread, or generate integrin tension on 56 pN TGT surfaces (Fig. S8, *B*–*E*). Thus, the effects of ST6Gal-I in this investigation do not appear to involve changes in N-glycan branching or binding of the N-glycans to a galectin lattice.

Our findings verify that sialylation of EGFR by ST6Gal-I affects its activity, signaling, and membrane retention. It is possible the increased EGFR activity could directly result from increased membrane persistence and/or reduced internalization by clathrin-mediated endocytosis (103). This reduced membrane clearance could in turn result in sustained EGFR kinase activation and maintenance of downstream signaling leading to continued signal amplification (104, 105). One hypothesis is that membrane retention is driven by destabilization of the EGFR dimer interface by ST6Gal-I sialylation. This might seem counterintuitive as classically, decreased dimerization leads to a loss of EGFR activity. However, in this proposed mechanism, destabilized EGFR dimers lead to incomplete autophosphorylation and do not trigger the negative feedback strongly associated with transient EGF-induced signaling (106). Lack of negative feedback can lead to sustained signaling following ligand binding, analogous to the ligands epiregulin and epigen, which destabilize EGFR dimers and cause sustained activity (68, 107, 108). Modifications at the dimer interface that impede stabilization could also result in reduced clustering of EGFR and reduced endocytic internalization (34, 109). Molecular dynamics (MD) simulations investigating glycosylation of key Asn residues on EGFR dimerization align with this “negative binding cooperativity” model (110). Sialylation adds an additional level of regulation, which potentially drives structural changes and sustained activation of downstream signaling through retention of EGFR at the cell surface (111).

There has been much work identifying genetic drivers and underlying biochemical mechanisms by which genes and signaling pathways drive tumor formation (10). Our results support the plausibility for ST6Gal-I directly influencing EGFR signaling to regulate cell mechanics, another key element regulating tumor cell response to the microenvironment. Mechanical changes associated in cells and in the underlying tissue structure influence cancer metastasis and formation of secondary tumors. Changes in cell mechanics impact cancer hallmarks and are crucial in understanding cancer biology and in identifying biomarkers and novel therapeutic strategies. Given the widespread impact of sialylation and the prognostic value of ST6Gal-I expression, an improved understanding of how ST6Gal-I–mediated sialylation alters cell mechanics may open the door to a new range of cancer therapeutics. The results presented here help bridge the mechanistic gap in the field, while demonstrating the potential value in oncogenic mechanosignaling as a therapeutic target.

## Experimental procedures

### Synthesis of TGT strands

Tension gauge tether top strand labeled with cRGDfK peptide, cyclo[Arg-Gly-Asp-D-Phe-Lys(PEG-PEG)] (Peptides International), was prepared by coupling NHS-azide (Thermo Fisher Scientific) with a copper-assisted cycloaddition reaction as previously described (22, 112). In brief, cRGDfK-azide and the alkyne-21-BHQ2 oligonucleotide was mixed at a 2:1 ratio (final concentrations ∼200 μM:100 μM) in 100 μl 1× PBS with 5 mM sodium ascorbate and 0.1 μM preformed Cu-THPTA. The mixture was incubated for a minimum of 4 h at room temperature (RT). Salts, organic solvents, and unreacted reactants were removed from the above mixture by P2 gel filtration and further purified by reverse-phase HPLC (solvent A was 0.1 M TEAA, solvent B was 100% MeCN; initial condition was 10% B with a gradient of 1%/min and flow rate of 1 ml/min).

Tension gauge tether bottom strand was coupled to Cy3B-NHS ester by nucleophilic substitution (22, 50). Briefly, either the 12 pN or 56 pN TGT strand (final concentration 100 μM) was mixed with 50 μg Cy3B-NHS ester (pre-dissolved in 10 μl DMSO) in 0.1 M sodium bicarbonate solution (final volume 100 μl, pH = 9). The reaction was incubated overnight at RT, and subjected to P2 gel filtration, followed by reverse-phase HPLC as described above. The absorbance spectra at 260 nm was used to determine the oligonucleotide concentrations using Nanodrop 2000 UV-Vis Spectrophotometer (Thermo Fisher Scientific). These purified tension sensors were characterized by MALDI-TOF mass spectrometry performed on a high-performance Voyager STR. The matrix for MALDI was prepared fresh at the time of experiment by dissolving excess 3-hydroxypicolinic acid into TA50 solvent (50:50 v/v acetonitrile and 0.1% TFA in ddH_2_O).

### Tension gauge tether surface preparation

The preparation of TGT surfaces was based on protocols described previously (22, 50, 52). Briefly, #2 coverslips (25 mm, VWR) were sonicated for 10 min in 200 proof alcohol (Decon Labs) and cleaned with piranha solution (3:1 H_2_SO_4_:H_2_O_2_, Thermo Fisher Scientific) for 30 min. Coverslips were washed with MilliQ water (6×) and ethanol (2×). Clean coverslips were then bonded with 3% (v/v) APTES (Sigma) in ethanol for 1 h, washed with ethanol (3×), and dried using N_2_ gas stream. The coverslips were then incubated overnight at 4 °C with 2 mg/ml sulfo-NHS-biotin in DMSO (Thermo Fisher Scientific). The following day, coverslips were washed with ethanol (3×) and dried with N_2_. To block nonspecific binding, surfaces were treated with 0.1% bovine serum albumin (BSA) (Thermo Fisher Scientific) in 1× PBS. Following washes with PBS (3×), the surfaces were treated with a solution of 1 μg/ml streptavidin (Thermo Fisher Scientific). After 45 min, the surfaces were washed with PBS (3×) and incubated for 1 h at RT with 100 μl of 50 nM preassembled DNA tension probes. Preassembly of tension probes was carried out in a thermocycler by incubating the probe mixture at 25 °C for 25 min after an initial denaturation for 5 min at 95 °C. Finally, the surfaces were washed with PBS (3×). Prior to imaging, Fluorobrite medium with or without EGF (Sigma) was introduced and the cells were added. Tension gauge tether surfaces were used within 24 h of synthesis.

### Cell culture and reagents

Cos-7 (African green monkey kidney fibroblast) cells were cultured in Dulbecco's modified Eagle's medium (DMEM) containing L-glutamine and sodium pyruvate (Corning) supplemented with 10% fetal bovine serum (FBS) and 100 IU/ml penicillin-streptomycin (PS) (Life Technologies). Stable polyclonal Cos-7 ST6Gal-I OE and EV cell lines were created by transducing WT Cos-7 cells with a ST6Gal-I lentiviral vector (Genecopoeia, cat # LPP-M0351-Lv105-200-S) or EV (Sigma) for 16 h at MOI 5. Virus-containing media was replaced with fresh complete media, and the cells were incubated for 48 h to allow sufficient puromycin-resistance gene expression. Transduced cells were selected with 10 μg/ml of puromycin (Sigma).

For ST3Gal-4 transduction, WT Cos-7 cells were transduced with ST3Gal-4 OE lentiviral vector (OriGene, cat # RC216090L3V) for 16 h at MOI 5 with 8 μg/ml of polybrene (Vector Builder) in serum-free media. Virus-containing media was replaced with fresh complete media and incubated for 48 h. Transduced cells were selected with 5 μg/ml of puromycin (Gibco, cat # A11138-03).

OVCAR3 cells were grown in RPMI media (Life Technologies) containing 20% FBS, 0.01 mg/ml bovine insulin (Life Technologies), and 100 IU/ml PS. OVCAR5 cells were grown in RPMI with 10% FBS and 100 IU/ml PS. OV4 cells were cultured in DMEM/Ham's F-12K 50:50 with 10% FBS and 100 IU/ml PS. OV4 cells were transduced with lentivirus encoding an EV (Sigma) or the human ST6Gal-I lentiviral vector (OE). OVCAR-3 and OVCAR-5 cells were transduced with a lentivirus containing either a short-hairpin ShC (Sigma) or shRNA against ST6Gal-I (KD) (Sigma, TRCN00000035432, sequence: CCGGCGTGTGCTACTACTACCAGAACTCGAGTTCTGGTAGTAGTAGCACACGTTTTTG) to knockdown ST6Gal-I expression. All transductions were performed using an MOI of 5. Stable polyclonal populations of cells were isolated with puromycin selection (5 μg/ml). Puromycin was removed for at least 2 days prior to experiments.

All cells were maintained at 37 °C and 5% CO_2_ and were validated to be negative for *mycoplasma* contamination.

Pharmacological inhibitors were diluted in DMSO and used at the following concentrations: Human TNFRI/TNFRSF1A Antibody, MAB625 (0.08 μg/ml; R&D Systems), BAY 11-7082, CAS 19542-67-7 (20 μM; Millipore Sigma), Anti-Fas Antibody (human, neutralizing), clone ZB4, 05-338 (500 ng/ml; Millipore Sigma), erlotinib HCl (20 nM; Selleckchem), ERK inhibitor SCH772984 (8 nM; Selleckchem), AKT inhibitor MK-2206 dihydrochloride (65 nM; Selleckchem), STAT inhibitor Niclosamide (1.4 μM; Selleckchem).

For the integrin-blocking experiments, the antibodies were diluted at 10 μl/ml in Fluorobrite DMEM. The antibodies used were as follows: Integrin β1 (D2E5) Rabbit mAb (Cell Signaling 9699), Integrin β3 (D7X3P) XP Rabbit mAb (Cell Signaling 13166), and Integrin β5 (D24A5) Rabbit mAb (Cell Signaling 3629).

### Epidermal growth factor stimulation

Epidermal growth factor was used at 50 ng/ml in all experiments and diluted in appropriate media (fluorobrite for TGT or DMEM for invasion, migration, proliferation, flow cytometry, and Western blot).

### Cell dissociation and stimulation on TGT surfaces

Confluent cells growing on a culture dish were washed in HBSS (Gibco; 14170-112) and dissociated with trypsin (Sigma). Residual trypsin was neutralized using a trypsin neutralizing buffer (Lonza; CC-5002) prior to plating cells on the TGT surfaces. Medium on the TGT surfaces was switched to Fluorobrite with or without 50 ng/ml EGF according to experimental conditions. For inhibitor studies, the cells were incubated in Fluorobrite supplemented with the indicated inhibitor for the entire incubation period.

### Immunostaining

Cos-7 cells were allowed to spread for 90 min on the TGT surface at 37 °C with 5% CO_2_. Following this, the cells were fixed in 4% (v/v) formaldehyde (Electron Microscopy Sciences) in 1× PBS for 15 min at 37 °C in a shaker with mild agitation (35 rpm). After neutralizing the fixative with washing (PBS, 5×), the cells were permeabilized (when staining for cytoplasmic proteins like F-actin, β-actin, or paxilling) and blocked for 30 min with 0.25% (v/v) Triton X-100 (Thermo Fisher Scientific) and 1% BSA. The cells were stained for the actin cytoskeleton using phalloidin, for FAs using paxillin, and for arginylated beta actin. For staining the surface EGFR, the EGF Receptor antibody was used. The dilutions for staining were as follows: paxillin (1:250; Abcam, ab32084), phalloidin (1:400; Cell Signaling, 8878s), anti-beta actin, arginylated (N-terminal – 2 μg/ml; Millipore Sigma, ABT264), EGF Receptor (D38B1) XP Rabbit mAb (1:200; Cell Signaling, 4267s), and Alexa Fluor-647 labeled goat anti-rabbit secondary antibody (1:800; Invitrogen, A-21244).

### Flow cytometry

To correlate changes in ST6Gal-I overexpression with alterations in cell surface α2,6 protein sialylation, cells were stained with the SNA lectin, which specifically binds α2,6 sialic acids. Accutase cell detachment solution (Biolegend; 423201) was used to suspend cells. The cells were first fixed for 20 min in 2% paraformaldehyde at RT. Following two washes with FACS buffer (1× PBS without Ca^2+^ and Mg^2+^, 5 mM EDTA, 1% BSA, 25 mM Hepes, 0.02% sodium azide), the cells were incubated for 30 min at RT with a 1:200 dilution of SNA Lectin conjugated to Cy5 (Novus Biologicals, CL-1305-NB). The cells were spun down and washed with FACS buffer and transferred to a flow tube. The BD LSR II flow cytometer (BD Biosciences) was used to analyze surface staining. Mean fluorescence intensity values were calculated using FlowJo. To confirm changes in cell surface EGFR, the cells were stained with 1:40 dilution of PE Anti-EGFR antibody (Abcam; ab130738). All other treatment steps were consistent.

### Microscopy

Cells were imaged on a Nikon Eclipse Ti2 microscope using the Nikon Elements software. The cells were imaged by TIRF and RICM with an oil immersion Apo TIRF 60× NA 1.49 objective and a cooled electron-multiplying charge-coupled device camera (iXon3; Andor Technology). The sample was illuminated with a Sola epifluorescence light source (Lumencor) for RICM or with 488, 561, or 638 nm lasers for TIRF.

### SNA precipitation assay

1000 μg of cell lysate was incubated with 200 μg of SNA-agarose (Vector Labs, AL-1303) at 4 °C overnight on a rotator. The bound proteins were precipitated by centrifugation and washed (3×) with ice cold PBS. Precipitates were resolved by SDS-PAGE and immunoblotted for EGFR as described below.

### Western blot analysis

Cells were lysed in radioimmunoprecipitation assay buffer (50 mM Tris–HCl, 150 mM NaCl, 1.0% (v/v) NP-40, 0.5% (w/v) sodium deoxycholate, 1.0 mM EDTA, 0.1% (w/v) SDS, and 0.01% (w/v) sodium azide at pH 7.4) with protease/phosphatase inhibitor cocktail (Complete, Roche). The lysates were clarified by centrifugation and protein concentration was determined with a bicinchoninic acid assay (Thermo Fisher Scientific). Fifty micrograms of protein was loaded per sample. GAPDH was used as a loading control. Images were captured with an Odyssey Image Station (Li-Cor), and the Odyssey Application Software (3.0, Li-Cor) was used for quantification. Primary antibodies were incubated overnight at 4 °C. Secondary antibodies were incubated for 30 min at RT. Primary antibodies used were as follows: ST6Gal-I (1 μg/ml; R&D Biosystems, AF5924), ST3Gal-4 (0.25 μg/ml; Invitrogen, PA5-42911), EGFR (D38B1) XP (1:1000; Cell Signaling, 4267), anti-phospho-EGFR (Tyr1068) (D7A5) XP (1:800; Cell Signaling, 3777), Phospho-p44/42 MAPK (Erk1/2) (Thr202/Tyr204) (D1314.4E) XP Rabbit mAb (1:2000; Cell Signaling, #4370), p44/42 MAPK (Erk1/2) Antibody (1:1000; Cell Signaling, #9102), Phospho-Akt (Ser473) (D9E) XP Rabbit mAb (1:2000; Cell Signaling, #4060), Akt (pan) (C67E7) Rabbit mAb (1:1000; Cell Signaling, #4691), Phospho-Stat3 (Tyr705) (D3A7) XP Rabbit mAb (1:2000; Cell Signaling, #9145), Stat3 (D1B2J) Rabbit mAb (1:1000; Cell Signaling, #30835), Integrin β1 (D2E5) Rabbit mAb (1:1000; Cell Signaling, #9699), Integrin β3 (D7X3P) XP Rabbit mAb (1:1000; Cell Signaling, 13166), Integrin β5 (D24A5) Rabbit mAb (1:1000; Cell Signaling, 3629), β-Tubulin (D3U1W) Mouse mAb (1:1000; Cell Signaling, #86298), and GAPDH (D4C6R) (1:1000; Cell Signaling, #97166). Secondary antibodies included Alexa Fluor 680-conjugated goat anti-rabbit (1:10,000; Life Technologies, A21109), goat anti-mouse (1:15,000; Li-Cor, 926-32210, IRDye 800CW), goat anti-rabbit (1:15,000; Li-Cor, 925-32211, IRDye 800CW), and goat anti-mouse (1:20,000; Li-Cor, 925-68020, IRDye 680LT).

### Transwell chamber migration and invasion assays

Cell migration and invasion was assessed by Transwell assays (Millipore Sigma, CLS-3422). All the cells were serum starved for 4 h prior to plating. For invasion assays, the transwell membrane was precoated on ice with 100 μl of Matrigel (Thermo Fisher, CB-40234A) and incubated at 37 °C for at least 2 h. Six hundred fifty microliters of corresponding media (50 ng/μl EGF in complete DMEM or serum free DMEM) was added to lower chamber. 1 × 10ˆ5 cells were seeded in the upper transwell chamber in 200 μl of serum-free media and incubated for 16 h (migration assay) or 24 h (invasion assay). Following incubation, the cells were removed from the top of the transwell membrane by cotton swabs. Transwells were then fixed and stained with 0.5% crystal violet in 25% methanol for 1 h. The transwells were than imaged at 40× on EVOS FL-Core system, and the cells were manually counted. Crystal violet was solubilized with 10% acetic acid for 15 min, and the absorbance was read at 590 nm using a Microplate reader (Biotek).

### 5-Bromo-2′-deoxyuridine assay

Cell proliferation was assessed by the 5-Bromo-2′-deoxyuridine (BrdU) assay which measured DNA synthesis by incorporation of the thymidine nucleotide analog BrdU (Thermo Fisher). Cos-7 cells (OE, EV, and WT) were seeded on coverslips in 6-well plates at a concentration of 1.5 × 10ˆ5. The cells were incubated with 10 μM BrdU solution for 2 h at 37 °C in the presence or absence of EGF. The cells were washed in PBS (3×) and fixed with 3% glyoxal for 10 min. The fixed cells were washed (3×) and permeabilized with 0.1% Triton X-100-PBS for 7 min. Following washing (6×), the DNA was hydrolyzed with 1 M HCl at RT for 30 min followed by blocking and immunostaining with an anti-BrdU monoclonal antibody (1:100; Thermo Fisher; MA3-071) for 2 h at RT. Finally, the cells were incubated with Alexa Fluor anti-mouse secondary for 30 min at RT (1:200; Thermo Fisher, A32728). Nuclei were stained with DAPI for 10 min at RT (Sigma, D9542). The cells were imaged on a Nikon Ti2 microscope using a 20× objective by collecting 10 fields of view per coverslip (n = 30 fields, across three independent experiments), and BrdU positive cells were manually counted.

### Sulforhodamine cell proliferation assay

Serum-starved Cos-7 cells (OE, EV, and WT) were seeded in a 96-well plate at a starting concentration of 5000 or 10,000 cells per well in the presence or absence of EGF. Three hours post-plating, the cells were fixed with 50% TCA at 4 °C for 1 h. The plates were washed 4× with H_2_O and stained with 0.04% Sulforhodamine B solution prepared in 1% acetic acid for 1 h at RT. The plates were washed (4×) with 1% acetic acid to remove any residual unbound dye and air dried. The cell-bound fraction was resuspended in 10 mM Tris base solution (pH 10.5) by agitating on an orbital shaker for 10 min at RT. The absorbance was measured at 510 nm using a microplate reader (Biotek).

### Clonogenic survival assay

Clonogenic survival was assessed by standard Colony Formation Assay. Cells were seeded in a 6-well plate at 100, 250, or 500 cells per well. The cells were treated with 100 ng/μl EGF and incubated for 2 weeks. The cells were then fixed and stained with 0.5% crystal violet in 25% methanol solution for 1 h. The plates were rinsed and colonies were counted.

### Image processing and statistical analysis

All images were analyzed and processed using Fiji (ImageJ, National Institutes of Health), Nikon Elements, and MATLAB. For consistency, the LUT were normalized to the same threshold limits representing the full dynamic range for all acquired images. Custom-written ImageJ macros were employed to subtract background fluorescence and measure morphological parameters, including area of the cell footprint (RICM area), circularity, and integrated tension. The RICM image was outlined manually to define the cell boundary which helps define the cell footprint for integrin tension calculations. Integrated tension was determined by calculating the total fluorescence intensity for all open TGT probes within the cell boundary and subtracting the background measured from an off-cell region. Tension gauge tether background corresponds to the fluorescence from quenched (unopened) TGT probes not experiencing any force.

Focal adhesion size and number was quantified as previously described (113). Briefly, the raw fluorescent images were background subtracted with a rolling ball radius set to 50 pixels. The local contrast of the image was enhanced by running the Contrast Limited Adaptive Histogram Equalization plugin. A mathematical exponential can then be applied (EXP) to further minimize the background. Next, the Laplacian of Gaussian filter was applied to blur the image to better identify FAs. Finally, the image was thresholded using Huang’s fuzzy thresholding method and analyzed using the Analyze Particles command to count and measure the size of FAs. For FA size analysis, puncta smaller than 0.2 μm^2^ were excluded from the analysis.

All results are presented as mean ± SD unless otherwise noted. Statistical calculations were performed using Prism6 software (GraphPad). One-way ANOVA was used to quantify the statistical significance.

## Data availability

All raw data is available upon request.

## Supporting information

This article contains supporting information.

## Conflict of interest

The authors declare that they have no conflicts of interest with the contents of this article.
