# Supplementary material for: ST6Gal-I–mediated sialylation of the epidermal growth factor receptor modulates cell mechanics and enhances invasion
Source: J Biol Chem. 2022 Feb 12;298(4):101726. doi: 10.1016/j.jbc.2022.101726 (PMC8956946; doi:10.1016/j.jbc.2022.101726)
Supplement: Supplemental Figures S1–S8 [file mmc1.pdf]

ST6Gal-I-mediated sialylation of the epidermal growth factor receptor modulates cell mechanics and enhances invasion

Tejeshwar C. Rao[1], Reena R. Beggs[1], Katherine E. Ankenbauer[1], Jihye Hwang[1], Victor Pui-Yan Ma[2], Khalid Salaita[2], Susan L. Bellis[1] and Alexa L. Mattheyses[1]

[1]Department of Cell, Developmental, and Integrative Biology, University of Alabama at Birmingham, Birmingham AL 35294

[2]Department of Chemistry, Emory University, Atlanta, Georgia 30322, USA

Supplementary Figures:

Figure S1. Schematic for TGT surface synthesis

Figure S2. ST6Gal-I regulates cell spreading, integrin tension and FA maturation irrespective of the underlying substrate tension threshold

Figure S3. ST6Gal-I dependent sialylation does not alter the sialylation profile of key integrin subtypes

Figure S4. ST3Gal-4 overexpression does not regulate cell spreading, integrin tension and FA maturation in an EGF-dependent manner

Figure S5. Analysis pipeline to evaluate ST6Gal-I OE dependent changes in the leading edge of a migratory cell with EGF stimulation

Figure S6. ST6Gal-I promotes mechanical tension primarily via ERK signaling on 12 pN TGTs

Figure S7. Validation of ST6Gal-I expression and α2,6 sialylation in OVCAR3, OVCAR5, and OV4 cell lines

Figure S8. MGAT5 does not influence ST6Gal-I mediated EGFR sialylation in Cos-7 cells

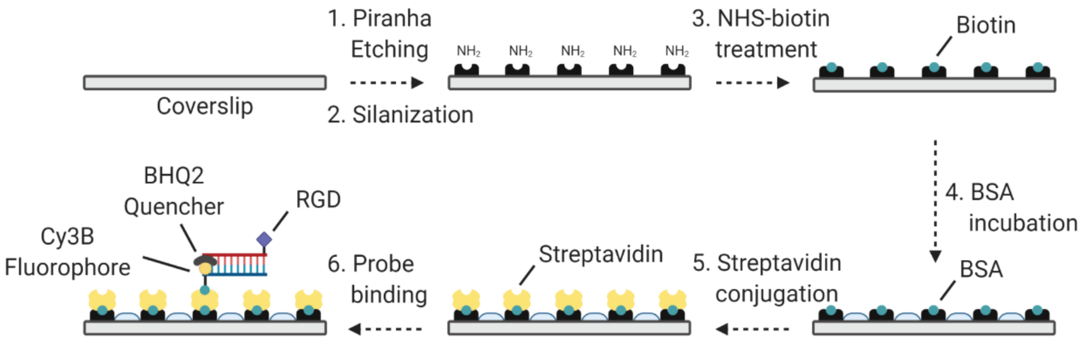

Figure S1. Schematic for TGT surface synthesis. Flowchart illustrating the fabrication process for the TGT-based tension surface (see methods for full description).

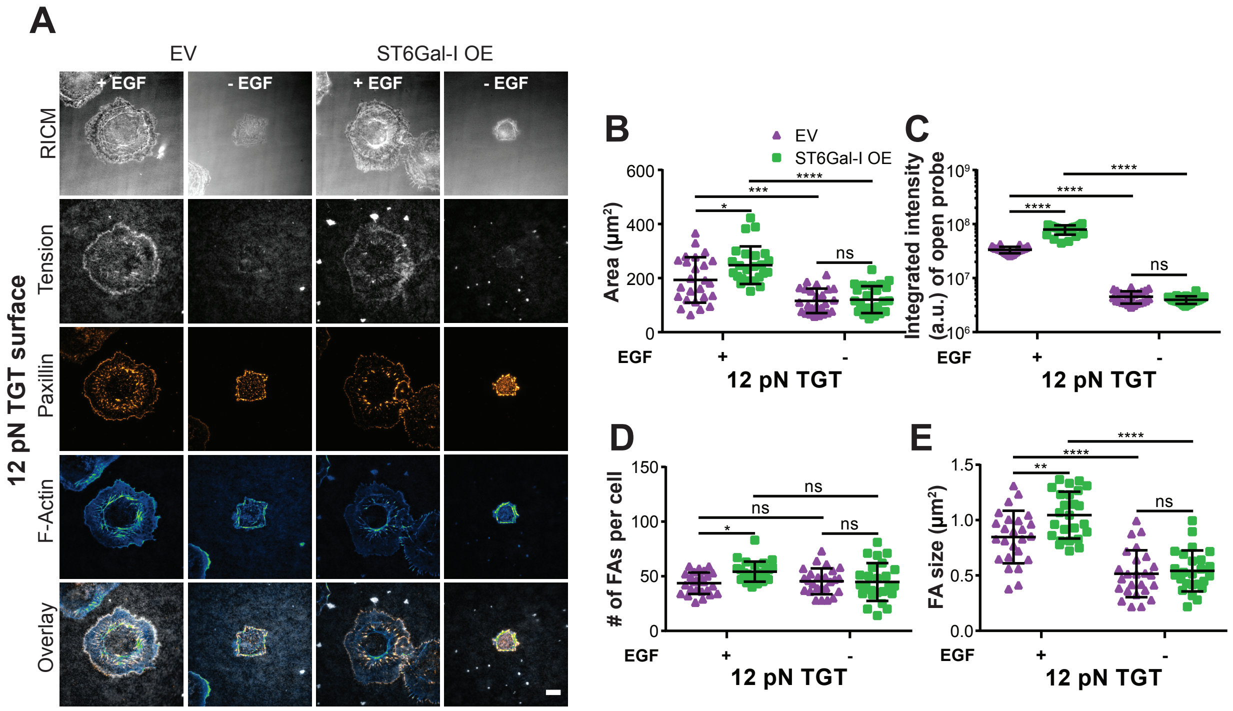

Figure S2. ST6Gal-I regulates cell spreading, integrin tension and FA maturation irrespective of the underlying substrate tension threshold. (**A**) Images of empty vector (EV) and ST6Gal-I OE Cos-7 cells on 12 pN TGT surfaces 90 mins post plating in the presence or absence of EGF (RICM, integrin tension - grayscale, paxillin - orange hot, and actin - green blue; scale bar = 10 µm). (**B-E**) Quantification of the (**B**) cell spread area (with EGF: EV, 193.3 ± 84.0 µm^2,OE, 247.6 ± 69.6 µm^2; without EGF: EV, 116.0 ± 45.1 µm^2, OE, 120.4 ± 49.6 µm^2), (**C**) integrated intensity of open probes (with EGF: EV, 4.0*10^7 ± 2.7*10^6 a.u., OE, 8.2*10^7 ± 3.2*10^6 a.u.; without EGF: EV, 8.6*10^6 ± 3.6*10^5 a.u., OE, 8.4*10^6 ± 2.4*10^5 a.u.), (**D**) number of FAs per cell (with EGF: EV, 43.6 ± 9.8, OE, 54.2 ± 9.2; without EGF: EV, 45.4 ± 11.8, OE, 44.8 ± 17.3), and (**E**) FA size (with EGF: EV, 0.85 ± 0.2 µm^2, OE, 1.04 ± 0.2 µm^2; without EGF: EV, 0.52 ± 0.2 µm^2, OE, 0.54 ± 0.2 µm^2). (mean ± SD, n = 25 cells across three independent experiments; $^{ns}p > 0.05$, *$p < 0.05$, **$p < 0.01$, ****$p < 0.0001$ by one-way ANOVA with Tukey's test).

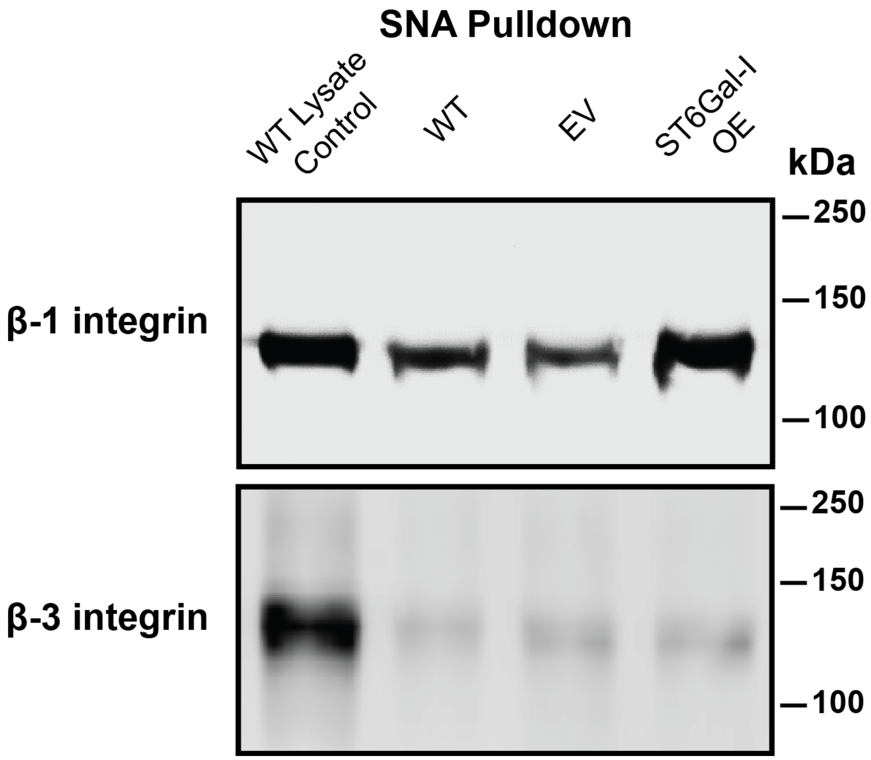

Figure S3. ST6Gal-I dependent sialylation does not alter the sialylation profile of key integrin subtypes. Representative immunoblots of β1 and β3 integrin following SNA pulldown from Cos-7 cells with ST6Gal-I overexpression (OE), empty vector (EV), and wildtype (WT) controls with EGF stimulation (10 min). WT cell lysate was used as a loading control.

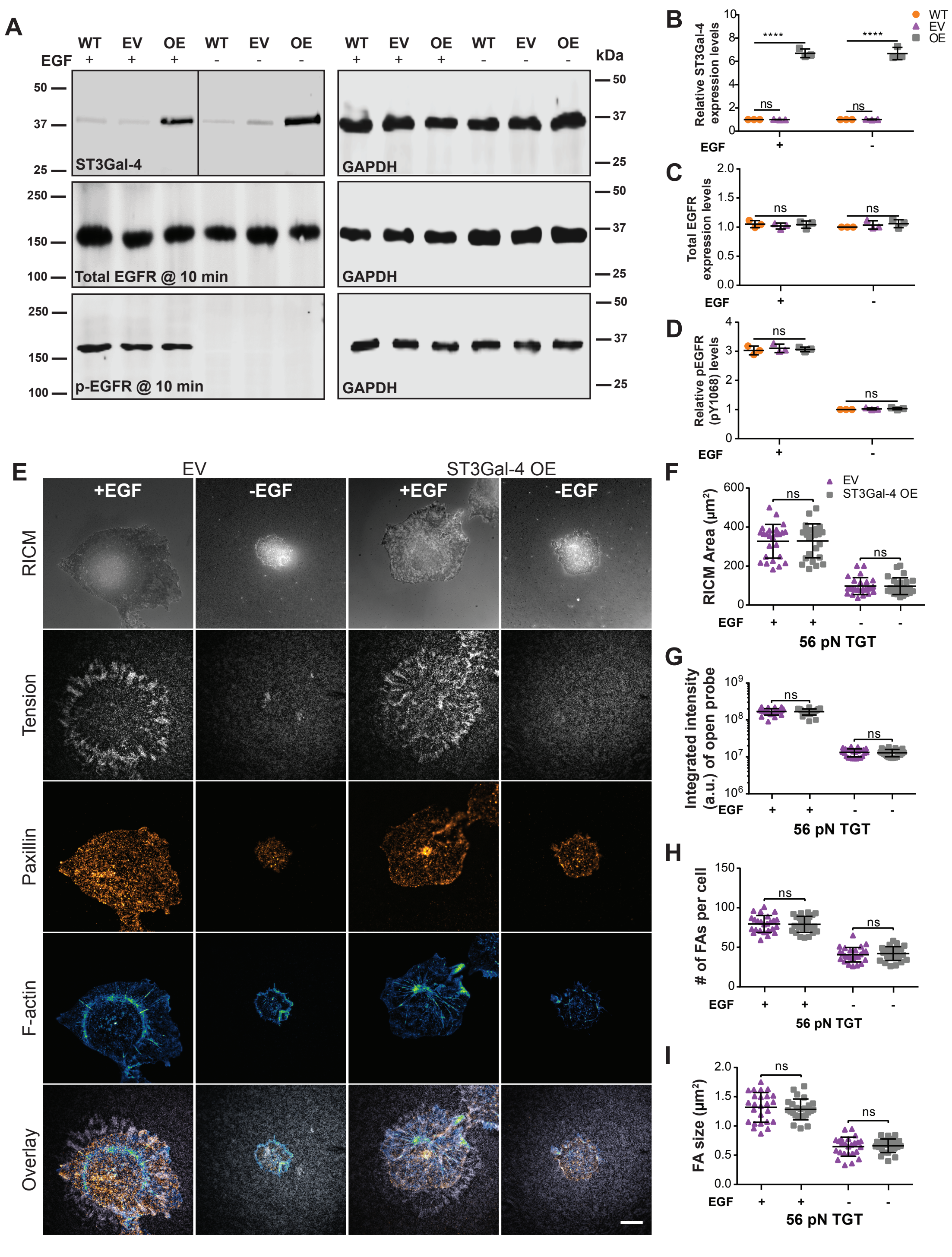

Figure S4. ST3Gal-4 overexpression does not regulate cell spreading, integrin tension and FA maturation in an EGF-dependent manner. (**A**) Representative immunoblots of ST3Gal-4, total EGFR, and pEGFR from Cos-7 cells stably transduced with lentivirus encoding human ST3Gal-4 (OE) or empty vector (EV) and wildtype (WT) controls with (10 min) or without EGF stimulation. GAPDH was used as the loading control. (**B-D**) Quantification of (**B**) ST3Gal-4, (**C**) total EGFR, and (**D**) p-EGFR normalized to WT cells without EGF treatment. (mean ± SD, n = 3 independent sets of

experiments; $^{ns}p > 0.05$, $^{*}p < 0.05$, $^{***}p < 0.001$, $^{****}p < 0.0001$ by one-way ANOVA with Tukey's test). (**E**) Images of empty vector (EV) and ST3Gal-4 overexpressing (OE) Cos-7 cells on a 56 pN TGT surface 90 mins post plating in the presence or absence of EGF (RICM, integrin tension - grayscale, paxillin - orange hot, and actin - green blue; scale bar = 10 μm). (**F-I**) Quantification of the (**F**) cell spread area (with EGF: EV, 327.0 ± 86.6 μm^2, OE, 329 ± 86.8 μm^2; without EGF: EV, 97.4 ± 43.4 μm^2, OE, 96.7 ± 43.1 μm^2), (**G**) integrated intensity of open probes (with EGF: EV, $1.7*10^8 \pm 3.4*10^7$ a.u., OE, $1.7*10^8 \pm 3.2*10^7$ a.u.; without EGF: EV, $1.3*10^7 \pm 3.3*10^6$ a.u., OE, $1.3*10^7 \pm 2.9*10^6$ a.u.), (**H**) number of focal adhesions (FAs) per cell (with EGF: EV, 79.5 ± 10.8, OE, 79.0 ± 10.2; without EGF: EV, 40.6 ± 9.3, OE, 42.0 ± 8.8), and (**I**) FA size (with EGF: EV - 1.32 ± 0.3 μm^2, OE, 1.28 ± 0.2 μm^2; without EGF: EV, 0.65 ± 0.2 μm^2, OE, 0.66 ± 0.1 μm^2) (mean ± SD, n = 25 cells across three independent experiments; $^{ns}p > 0.05$, $^{*}p < 0.05$, $^{**}p < 0.01$, $^{****}p < 0.0001$ by one-way ANOVA with Tukey's test).

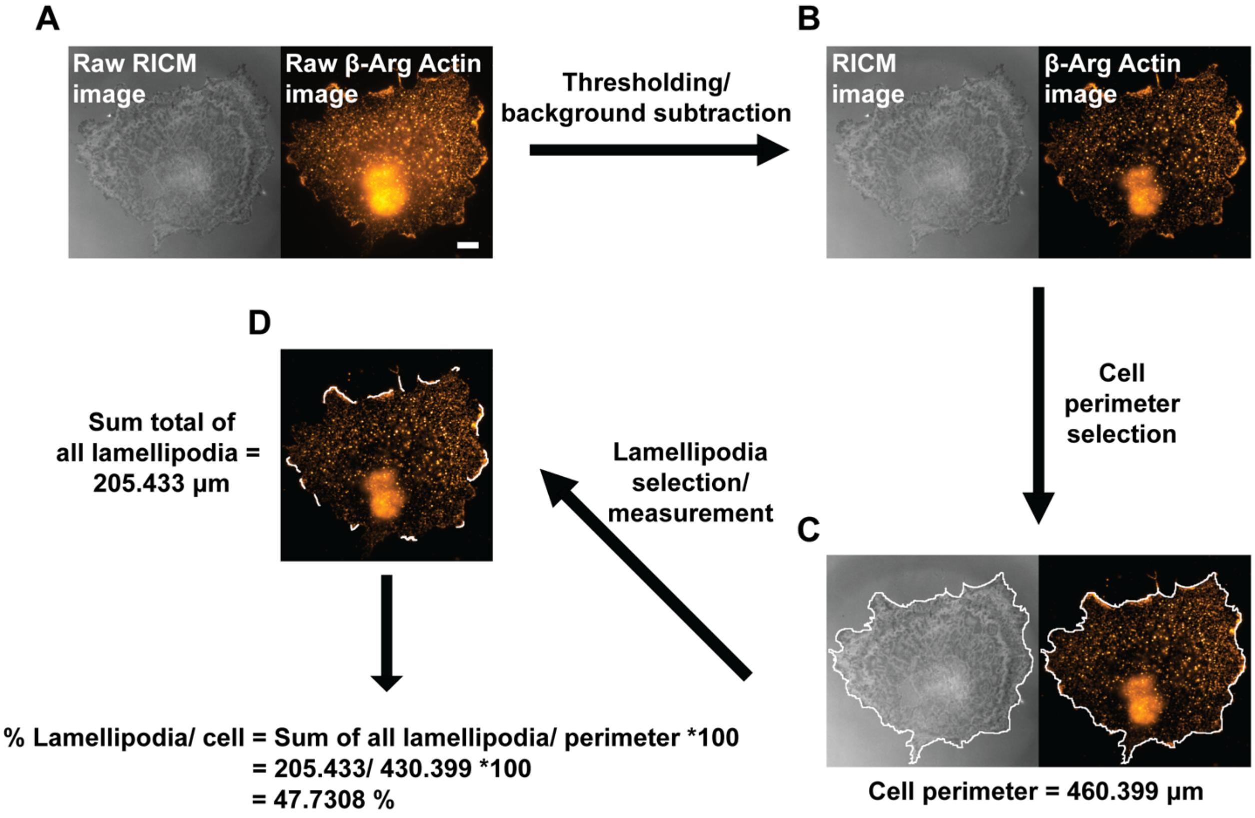

Figure S5. Analysis pipeline to evaluate ST6Gal-I OE dependent changes in the leading edge of a migratory cell with EGF stimulation. The pipeline is demonstrated with the representative ST6Gal-I OE Cos-7 cell from Figure 5A. **(A)** Representative ST6Gal-I OE Cos-7 cell with images of the cell footprint (RICM) and β-arginylated actin (TIRF) (scale bar = 10 µm) (**B**) Images from (**A**) after thresholding and background subtraction. (**C**) The cell perimeter was outlined manually using the RICM image as a reference to define the cell boundary. (**D**) The lamellipodia edge with β-arginylated actin was selected using the TIRF image. The percentage lamellipodia per cell was calculated as a ratio of the perimeter positive for lamellipodia over the total cell perimeter multiplied by 100.

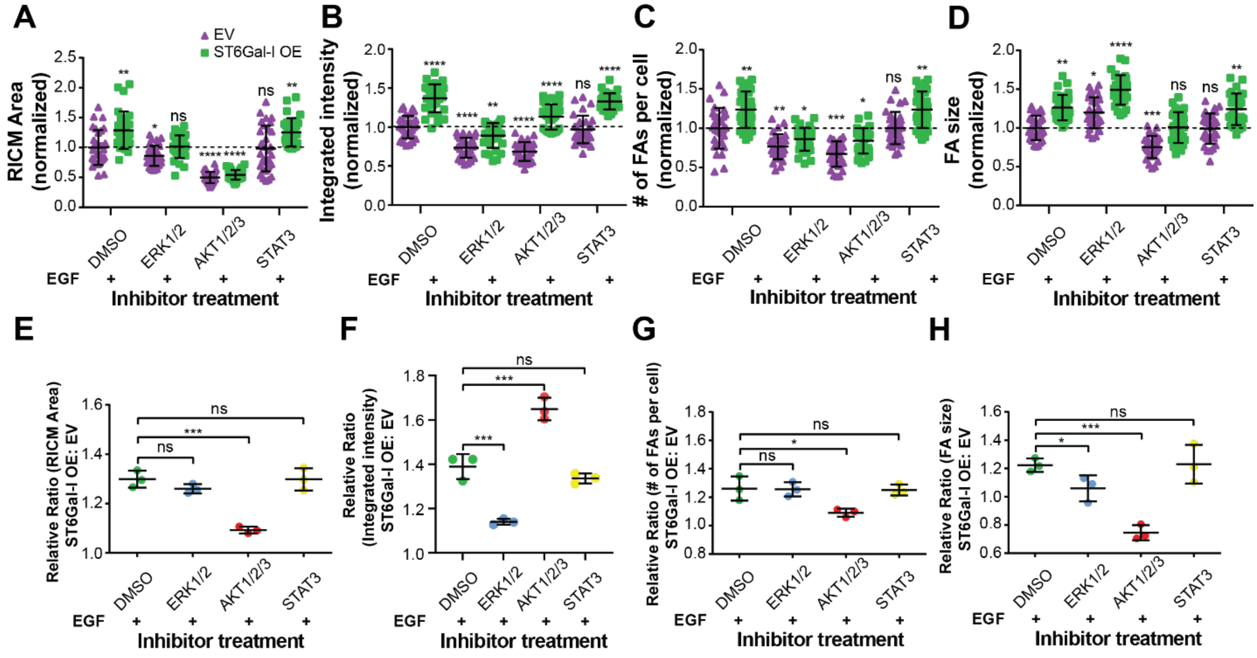

Figure S6. ST6Gal-I promotes mechanical tension primarily via ERK signaling on 12 pN TGTs. ST6Gal-I OE and EV Cos-7 cells were treated with control (DMSO) Erk, Akt, or STAT inhibitors with EGF on a 12 pN TGT surface, fixed and imaged 90 mins post plating. (**A-D**) Scatter plots normalized to control treated EV cells of the (**A**) RICM area (EV: DMSO, 1.00 ± 0.29; ERK, 0.86 ± 0.17; AKT, 0.49 ± 0.09; STAT, 0.98 ± 0.38; OE: DMSO, 1.29 ± 0.31; ERK, 1.01 ± 0.19; AKT, 0.54 ± 0.08; STAT, 1.25 ± 0.24), (**B**) integrated intensity of open probes (EV: DMSO, 1.00 ± 0.15; ERK, 0.73 ± 0.13; AKT, 0.69 ± 0.12; STAT, 0.97 ± 0.18; OE: DMSO, 1.37 ± 0.18; ERK, 0.89 ± 0.16; AKT, 1.13 ± 0.16; STAT, 1.33 ± 0.10), (**C**) number of focal adhesions (FAs) per cell (EV: DMSO, 1.00 ± 0.26; ERK, 0.76 ± 0.16; AKT, 0.67 ± 0.16; STAT, 1.01 ± 0.20; OE: DMSO, 1.24 ± 0.23, ERK, 0.86 ± 0.15; AKT, 0.84 ± 0.16; STAT, 1.24 ± 0.23), and (**D**) FA size (EV: DMSO, 1.00 ± 0.16; ERK, 1.20 ± 0.19; AKT, 0.75 ± 0.14; STAT, 0.99 ± 0.19; OE: DMSO, 1.26 ± 0.16; ERK, 1.49 ± 0.19; AKT, 1.01 ± 0.20; STAT, 1.24 ± 0.20). (**E-H**) Cumulative plots showing the relative ST6Gal-I OE to EV ratio for the morphometric and mechanical outcomes for each experimental set measured above: (**E**) RICM area (DMSO, 1.30 ± 0.03; ERK, 1.26 ± 0.02; AKT, 1.09 ± 0.01; STAT, 1.30 ± 0.04), (**F**) integrated intensity of open probes (DMSO, 1.39 ± 0.04; ERK, 1.14 ± 0.01; AKT, 1.65 ± 0.04; STAT, 1.32 ± 0.04), (**G**) number of FAs per cell (DMSO, 1.26 ± 0.09; ERK, 1.26 ± 0.05; AKT, 1.09 ± 0.03; STAT, 1.25 ± 0.04), and (**H**) FA size (DMSO, 1.22 ± 0.05; ERK, 1.06 ± 0.09; AKT, 0.75 ± 0.05; STAT, 1.23 ± 0.14). (mean ± SD; n = 25 cells across three independent experiments; $^{ns}p > 0.05$, $^{*}p < 0.05$, $^{**}p < 0.01$, $^{****}p < 0.0001$ by one-way ANOVA with Tukey's test).

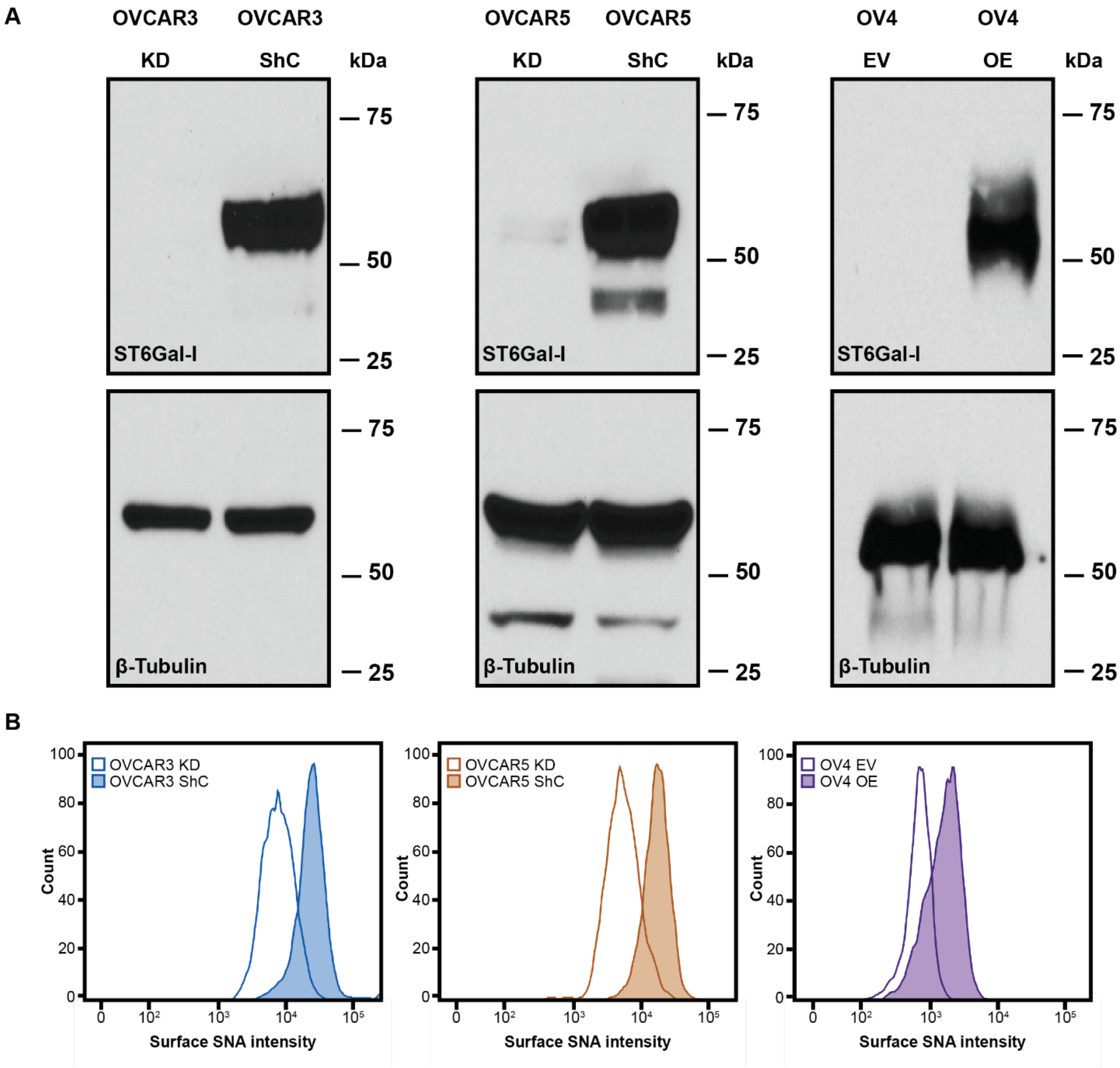

Figure S7. Validation of ST6Gal-I expression and α2,6 sialylation in OVCAR3, OVCAR5, and OV4 cell lines. (A) Representative immunoblots for ST6Gal-I, from OVCAR3 and OVCAR5 cells stably transduced with shRNA control (ShC), or shRNA against ST6Gal-1 (KD) and OV4 cells transduced with ST6Gal-I (OE) or empty vector (EV) lentivirus. (**B**) SNA staining of α2,6 surface sialylation in OVCAR3 (ShC and KD), OVCAR 5 (ShC and KD) and OV4 (EV and OE) cells as detected by flow cytometry.

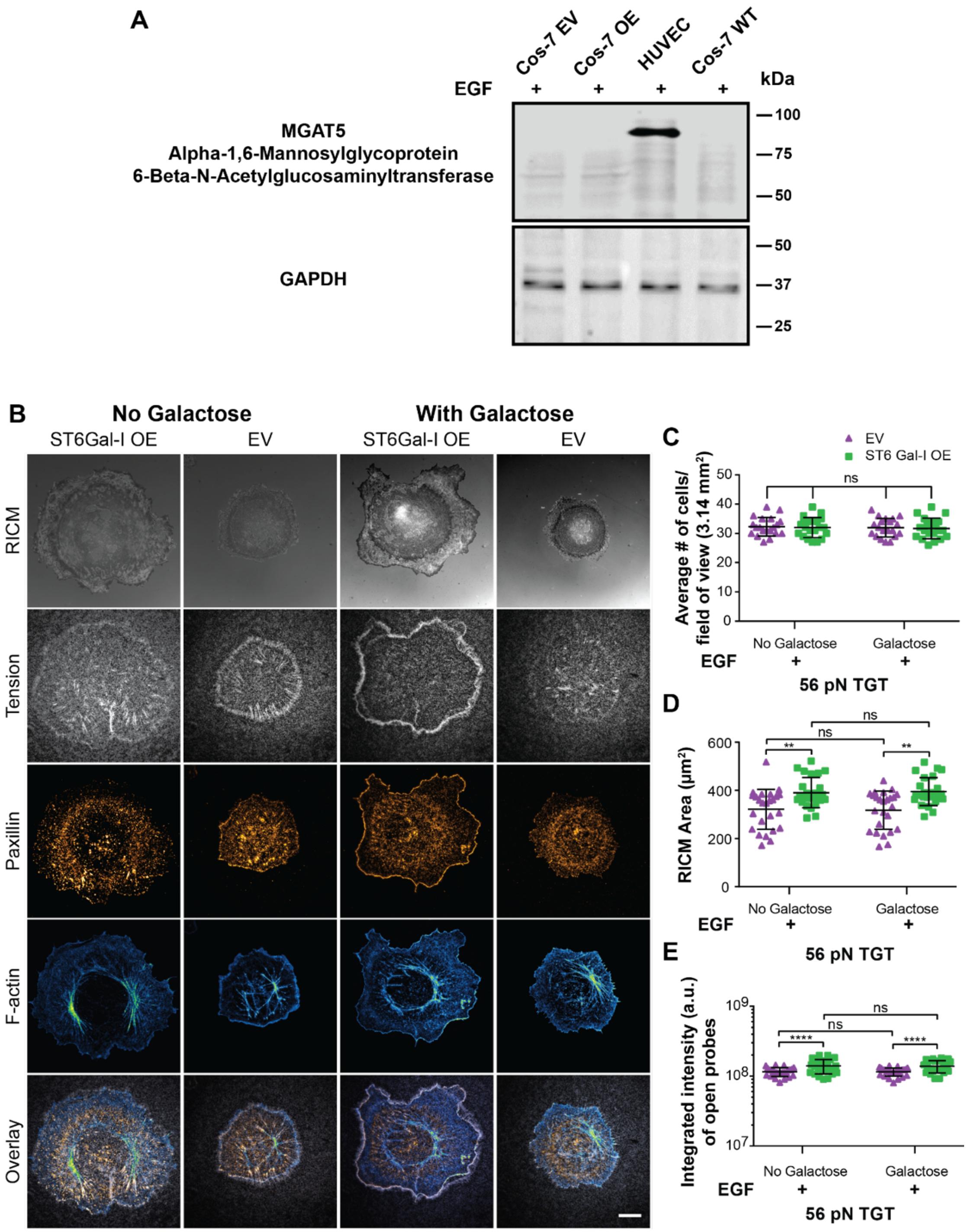

Figure S8. MGAT5 does not influence ST6Gal-I mediated EGFR sialylation in Cos-7 cells. (**A**) Representative immunoblots of MGAT5 from WT, EV and ST6Gal-I OE Cos-7 cells with (10 min) EGF stimulation. Human umbilical vein endothelial cell (HUVEC) lysate was used as a positive control. GAPDH was used as the loading control. (**B**) Images of empty vector (EV) and ST6Gal-I overexpressing (OE) Cos-7 cells on a 56 pN TGT surface 90 mins post plating in the presence of EGF with or without galactose pretreatment (RICM - raw image, integrin tension - grayscale, paxillin - orange hot, and actin - green blue; scale bar = 10 µm). (**C-E**) Quantification of the (**C**) average number of cells per field of

view (without galactose: EV, 32 ± 3.1; OE, 32 ± 3.4; with galactose: EV, 32 ± 3.2; OE, 32 ± 3.5), (**D**) cell spread area (without galactose: EV, 321.6 ± 83.0 μm^2; OE, 390.8 ± 62.7 μm^2; with galactose: EV, 317.9 ± 79.1 μm^2; OE, 395.0 ± 57.8 μm^2), and (**E**) integrated intensity of open probes (without galactose: EV, $1.2*10^8 \pm 1.6*10^7$ a.u.; OE, $1.40*10^8 \pm 3.26*10^7$ a.u.; with galactose: EV, $1.2*10^8 \pm 1.6*10^7$ a.u.; OE, $1.4*10^8 \pm 2.7*10^7$ a.u.) (mean ± SD, n = 25 cells across three independent experiments; $^{ns}p > 0.05$, $^{**}p < 0.01$, $^{****}p < 0.0001$ by one-way ANOVA with Tukey's test).
